# Structural Variations Associated with Adaptation and Coat Color in Qinghai‐Tibetan Plateau Cattle

**DOI:** 10.1002/advs.202503258

**Published:** 2025-06-05

**Authors:** Xiaoting Xia, Fuwen Wang, Xiaoyu Luo, Shuang Li, Yang Lyu, Yining Zheng, Zhijie Ma, Kaixing Qu, Rende Song, Jianyong Liu, Jicai Zhang, Basang Wangdui, Basang Zhuzha, Suolang Quji, Li Zhao, Silang Wangmu, Ciren Luobu, Nima Cangjue, Danzeng Luosang, Suolang Sizhu, Haijian Cheng, Ruizhe Li, Zhipeng Wu, Ruihua Dang, Yongzhen Huang, Xianyong Lan, Luohao Xu, Haifei Hu, WaiYee Low, Zhuqing Zheng, Yu Wang, Yuanpeng Gao, Lu Deng, Johannes A. Lenstra, Jianlin Han, Xueyi Yang, Wenfa Lyu, Bizhi Huang, Chuzhao Lei, Ningbo Chen

**Affiliations:** ^1^ Key Laboratory of Animal Genetics Breeding and Reproduction of Shaanxi Province College of Animal Science and Technology Northwest A&F University Yangling 712100 China; ^2^ State Key Laboratory of Plateau Ecology and Agriculture Qinghai University Xining 810016 China; ^3^ Key Laboratory of Animal Production Product Quality and Security Ministry of Education College of Animal Science and Technology Jilin Agricultural University Changchun 130118 China; ^4^ Qinghai Academy of Animal Science and Veterinary Medicine Qinghai University Xining 810016 China; ^5^ Academy of Science and Technology Chuxiong Normal University Chuxiong 675099 China; ^6^ Animal Disease Prevention and Control Centre of Yushu Tibetan Autonomous Prefecture Yushu 810099 China; ^7^ Yunnan Academy of Grassland and Animal Science Kunming 650500 China; ^8^ Institute of Animal Husbandry and Veterinary Science Tibet Academy of Agricultural and Animal Husbandry Sciences Lhasa 850030 China; ^9^ Animal Science College Tibet Agriculture and Animal Husbandry University Nyingchi 860000 China; ^10^ Institute of Animal Science and Veterinary Medicine Shandong Academy of Agricultural Sciences Shandong Key Lab of Animal Disease Control and Breeding Jinan 271018 China; ^11^ Key Laboratory of Aquatic Science of Chongqing College of Life Sciences Southwest University Chongqing 400715 China; ^12^ Rice Research Institute Guangdong Key Laboratory of New Technology in Rice Breeding and Guangdong Rice Engineering Laboratory Guangdong Academy of Agricultural Sciences Guangzhou 510640 China; ^13^ The Davies Research Centre School of Animal and Veterinary Sciences University of Adelaide Roseworthy SA 5371 Australia; ^14^ College of Computing and Information Technology University of Doha for Science and Technology Doha 00974 Qatar; ^15^ College of Food and Biology Jingchu University of Technology Jingmen Hubei 448000 China; ^16^ College of Veterinary Medicine Northwest A&F University Yangling 712100 China; ^17^ Faculty of Veterinary Medicine Utrecht University Utrecht NL 7758 The Netherlands; ^18^ Yazhouwan National Laboratory Sanya 572024 China; ^19^ Life Science College Luoyang Normal University Luoyang 471934 China

**Keywords:** cattle, coat color, genome assembly, high‐altitude adaptation, structural variation

## Abstract

Structural variations (SVs) play crucial roles in the evolutionary adaptation of domesticated animals to natural and human‐controlled environments, but SVs have not been explored in Tibetan cattle, which recently migrated and rapidly adapted to the high altitudes of the Qinghai‐Tibetan Plateau (QTP). In this study, a *de novo* chromosome‐level genome assembly for Tibetan cattle is constructed. It is found that using a lineage‐specific reference genome significantly increased variant detection accuracy and completeness. Analysis of long‐read sequencing data from 36 high‐altitude QTP and 48 low‐altitude cattle identified 222 528 SVs and 259 SV hotspot regions. Positively selected SVs in high‐altitude cattle are related to energy metabolism erythropoiesis and angiogenesis, and peroxisomal metabolism. A 102‐bp intronic deletion in *GNPAT* likely upregulated its expression. It is distinguished 7293 SVs that may be introgressed from yak, including variants upstream of the hypoxia‐inducing gene *EGLN1*. Finally, a ≈2‐Mb heterozygous inversion and two translocations on chromosome 6 are likely associated with the cattle gray coat via regulatory effects on the *KIT* gene. The results confirm the importance of SVs in evolutionary adaptation and the contribution yak‐introgressed SVs to the rapid acclimatization of QTP cattle.

## Graphical abstract

This study reveals the landscape of structural variants in Qinghai‐Tibetan Plateau cattle through long‐read sequencing. Discoveries include metabolic and oxygen‐regulation gene variants, along with a 2‐Mb *KIT*‐containing inversion and translocations responsible for cattle gray coat. These findings highlight the significant role of structural variants in the evolutionary adaptation of cattle on the Qinghai‐Tibetan Plateau.

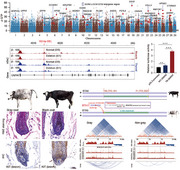

## 1 Introduction

The Qinghai‐Tibetan Plateau (QTP) is one of the most extreme environments on Earth, with an average altitude of 4268 m above sea level. Species that inhabit the QTP must adapt to harsh conditions, including low oxygen concentrations, high‐intensity UV radiation, and large fluctuations in temperature. The geographical range of the QTP includes the high mountain and plateau region in southwestern China (Xizang Autonomous Region, most of Qinghai, parts of Sichuan, Gansu, and Yunnan Province) and extends to neighboring countries. Several studies have been carried out to understand the adaptations of yak^[^ ^1^ ^]^ dog,^[^ ^2^, ^3^ ^]^ horse,^[^ ^4^ ^]^ pig,^[^ ^5^ ^]^ and chicken^[^ ^6^ ^]^ to high‐altitude environments. Interestingly, selection signatures suggest diverse evolution pathways underlying their adaptive selection. For example, *EPAS1*, a key gene involved in hypoxia adaptation, has undergone convergent selection in Tibetan human, horse, dog, and gray wolf.^[^ ^2^, ^4^, ^7^, ^8^, ^9^ ^]^ However, in yak and chicken, adaptation to high altitudes is achieved through the selection of other genes (e.g., *ADAM17* and *RYR2*).^[^ ^1^, ^6^ ^]^

The domestication of *Bos taurus* (taurine cattle) and *B. indicus* (indicine cattle) dates back more than 10 000 and 8000 years, respectively.^[^ ^10^, ^11^ ^]^ During the history and development of human society, cattle have spread and adapted to a vast variety of environmental conditions and have also experienced admixture with other bovine species.^[^ ^12^, ^13^, ^14^, ^15^, ^16^ ^]^ QTP cattle include mainly Tibetan taurine and indicine cattle. Ancient DNA evidence indicates that taurine cattle arrived on the QTP at least 3900 years ago and represent the earliest domesticated cattle entering East Asia.^[^ ^17^ ^]^ Tibetan cattle live at altitudes of 3000–4600 m and retain distinct morphological and other characteristics,^[^ ^13^ ^]^ such as small body size, thick coat, low metabolic rate, and strong adaptability to high altitudes. High‐altitude indicine cattle include mainly the Shigatse Humped cattle breed in Xizang Autonomous Region (≈4000 m), whereas molecular data show a clear indicine influence on the humpless Diqing cattle in Yunnan Province (≈3460 m).^[^ ^18^, ^19^ ^]^

Structural variations (SVs) are believed to have a broader influence on gene regulation and protein functionality than single nucleotide polymorphisms (SNPs) and shorter insertions or deletions (InDels) and are therefore also more important for understanding certain aspects of the adaptation process.^[^ ^20^, ^21^, ^22^, ^23^, ^24^ ^]^ Previous studies have used SNPs to identify selection signals or detect adaptative introgression from yak into Tibetan taurine cattle: this revealed several candidate genes related to the hypoxia‐inducible transcription factor pathway, including *EGLN1*.^[^ ^13^, ^14^, ^19^ ^]^ A recent study utilizing large‐scale genomic data revealed selected genes related to body size (*HMGA2* and *NCAPG*) and energy metabolism (*DUOXA2*) in Tibetan cattle.^[^ ^19^ ^]^ However, research on the adaptation and phenotypic associations of QTP cattle through SVs has not yet been conducted.

**Figure 1:**
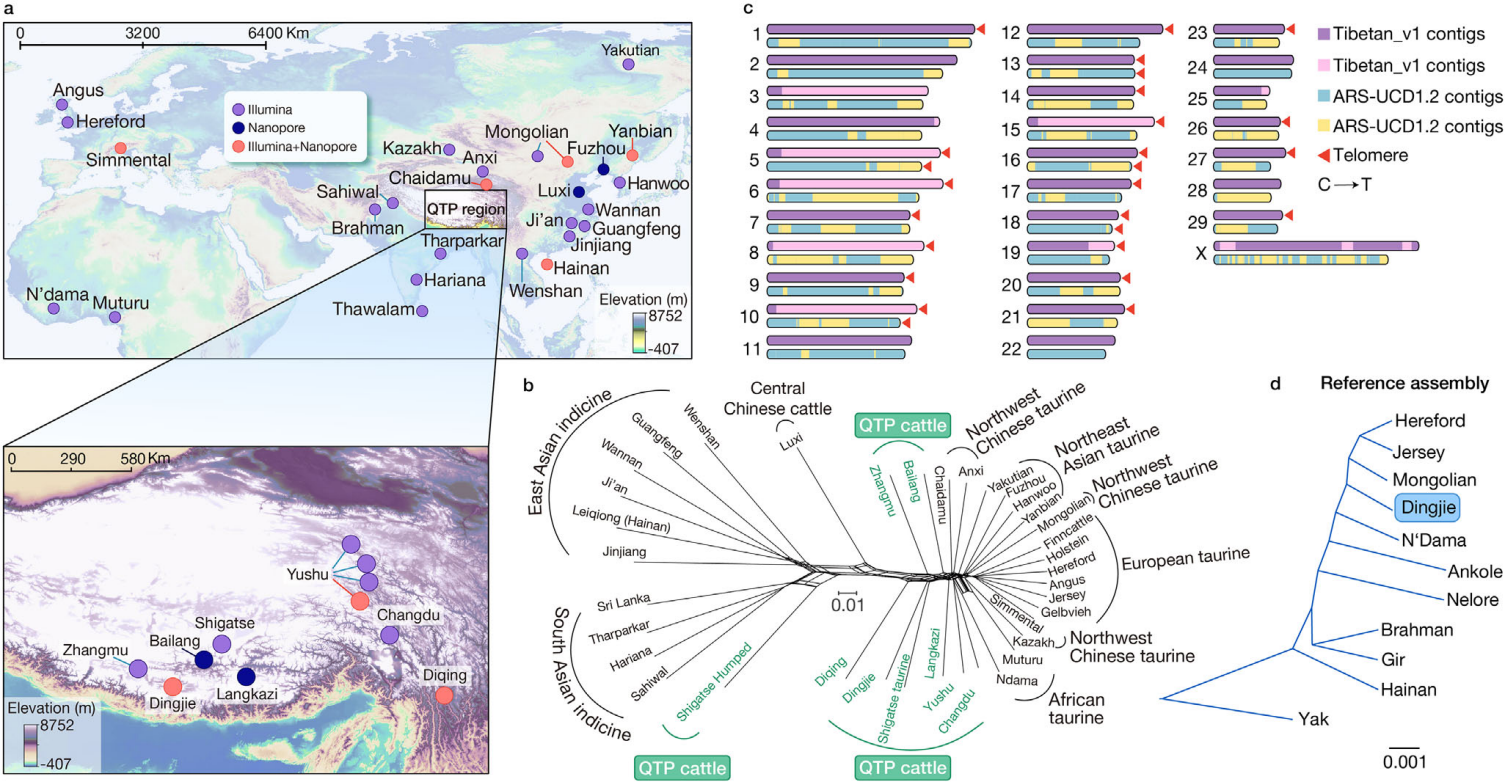
Geographic distribution, phylogenetic relationships, and genome assembly of Qinghai‐Tibetan Plateau cattle. a) Geographic locations of the cattle breeds used in this study and their sequencing platforms. b) NeighborNet graph of 37 cattle populations. An allele frequency‐dependent distance metric (Reynolds) was used to construct NeighborNet. c) Chromosome diagrams for the ARS‐UCD1.2 (bottom) and Tibetan_v1 (top) assemblies for autosomes from the centromere (left) to the telomere (right); the X chromosome has two telomeres. The positions of the telomeres are marked with orange triangles. d) A phylogenetic tree of MASH distances derived from 11 bovine assemblies, including the taurine cattle reference genome. The yak assembly was used as the outgroup.

Here, we generated a high‐quality *de novo* genome assembly of Tibetan cattle based on the Pacific Biosciences high‐fidelity (HiFi) long‐read and high‐throughput chromosome conformation capture (Hi‐C) data. We detected the SVs associated with high‐altitude adaptation by collecting long‐read sequencing (LRS) data from 84 cattle across various altitudes, including 36 cattle on the QTP at altitudes above 3000 m, specifically from the Xizang Autonomous Region, Qinghai, and Yunnan Province in China (**Figure** **1**a). To expand the sample size, these SVs were further genotyped in a larger panel of short‐read sequencing (SRS) data of 281 cattle and 12 individuals from other bovine species. Combined with epigenomic analysis, we highlight the unique contributions of SVs to high‐altitude adaptation and gray coat color in QTP cattle.

## 2 Results

### 2.1 Phylogenomic Relationships and *De Novo* Genome Assembly of QTP Cattle

To describe the population genetic structure of the QTP cattle populations, we generated an SNP dataset using 84 LRS and 281 SRS samples, which includes data from 148 QTP cattle (36 LRS and 112 SRS data) encompassing Changdu, Dingjie, Langkazi, Diqing, Yushu, Zhangmu, and Shigatse populations living at different altitudes (Figure 1a). A phylogenetic network (Figure 1b) revealed that QTP taurine cattle are closely related to Northeast Asian taurine and Northwest Chinese taurine cattle, whereas QTP indicine cattle (Shigatse Humped) are close to South Asian indicine cattle. The Diqing cattle exhibited mixed taurine‐indicine ancestry. These results were confirmed by phylogenetic tree of individuals (Figure S1a, Supporting Information) and principal component analysis (PCA, Figure S1b, Supporting Information).

We selected a male Tibetan cattle (Dingjie DJ12) living at an altitude of 4610 m to generate a Tibetan cattle reference genome sequence (Tibetan_v1). Assembly of 102.0 Gb of HiFi data by the Hifiasm program^[^ ^25^ ^]^ (Table S1 and Figure S2, Supporting Information) resulted in 709 contigs with an N50 of 84.6 Mb. After the incorporation of 188.5 Gb Hi‐C data, we obtained a genome of 2.92 Gbp, consisting of 31 chromosomes in 60 scaffolds, with a scaffold N50 of 110.9 Mb. The chromosomes of Tibetan_v1 are up to 24.50 Mb longer than those of the taurine cattle reference genome (ARS‐UCD1.2) (Figure 1c and Table S2, Supporting Information), which, for a large part (49%) was due to additional repetitive regions. We detected telomere sequences (TTAGGG)_n_ on 21 autosomes of Tibetan_v1 (ARS‐UCD1.2: 5 autosomes) (Figure 1c). A phylogenetic tree based on the Mash distances^[^ ^26^ ^]^ between genomes positioned Dingjie (Tibetan_v1) between the Mongolian (Eurasian taurine) and N'Dama (African taurine) genomes^[^ ^13^ ^]^ (Figure 1d).

An alignment of the Tibetan_v1 scaffolds against ARS‐UCD1.2 revealed high collinearity with the Tibetan_v1 genome (Figure S3, Supporting Information). SVs are nonrandomly distributed throughout the genome but are highly prevalent in the terminal regions of chromosomes (Figure S4, Supporting Information). Benchmarking Universal Single‐Copy Orthologs (BUSCO) evaluation revealed that 93.4% of the complete single‐copy genes in Tibetan_v1 were assembled out of the 9226 orthologous single‐copy genes (Table S3, Supporting Information). A *K*‐mer evaluation of the quality by the Merqury program^[^ ^27^ ^]^ gave a consensus quality score (QV) of Tibetan_v1 of 52.67, which was clearly higher than that of the other references (31.1 to 35.9), confirming that the Tibetan_v1 assembly is more complete than other cattle reference genomes (**Table** **1**). Interspersed repeats accounted for 44.25% of Tibetan_v1, among which long interspersed nuclear elements (LINEs) constituted the greatest proportion (26.15%), whereas short interspersed nuclear elements (SINEs) accounted for 10.88% (Table S4, Supporting Information).

**Table 1:** Comparison of the Tibetan_v1 *de novo* assembly with representative cattle assemblies.

Statistics	Tibetan_v1	Hainan_v1	Mongolian_v1	ARS‐UCD1.2
Genome size (bp)	2919463950	2651892370	2638491090	2715853794
Contig N50 (bp)	84618144	44352259	47790958	25896116
Scaffold N50 (bp)	110861251	104224000	104053164	103308737
Number of scaffolds	60	301	400	2211
Max scaffold (bp)	163509520	157081543	146429245	158534110
Min scaffold (bp)	21316	749	1000	1034
Scaffold mean (bp)	48657732	8810273	6596227	1228337
Consensus quality score (QV)	52.7	35.9	39.9	31.1
*K*‐mer based error rate	5.4 × 10^−6^	2.6 × 10^−4^	1.0 × 10^−4^	7.8 × 10^−4^
*K*‐mer completeness (%)	94.4	91.7	93.9	96.7

### 2.2 Application of Lineage‐specific Assembly in Population Genetic Analysis

We aligned the SRS data of Tibetan_v1 to each of the four assemblies (ARS‐UCD1.2, Tibetan_v1, Hainan_v1, and Mongolian_v1). As expected, Tibetan_v1 had the highest mapping rate (Table S5, Supporting Information). We then identified small variants by aligning the SRS data of 27 Dingjie cattle to ARS‐UCD1.2 and Tibetan_v1 reference genomes. Alignments to Tibetan_v1 resulted in more singleton and fixed variants than alignment to ARS‐UCD1.2 (Figures S5a,b, Supporting Information), indicating that lineage‐specific assembly facilitates the discovery of rare variants. The use of a lineage‐specific reference could convert “multiallelic” into “biallelic” loci in a target lineage.^[^ ^28^ ^]^ We found that from the multiallelic variants identified in the 27 Dingjie cattle against the ARS‐UCD1.2, 5.58–8.41% (708–1417 per genome) of them were converted into biallelic variants by alignment to Tibetan_v1 (Table S6, Supporting Information), resulting in 11928 biallelic variants linked with 5056 genes. Among them, 265 SNPs were found in exons of 181 genes. These genes were significantly enriched in pathways related to immunity (corrected *P* value < 0.05), which might be important for the adaptation of Tibetan cattle to high altitudes^[^ ^13^ ^]^ (Table S7 and Figure S5c, Supporting Information). This example indicates that calling for variants based on non‐lineage‐specific references may miss important variants. However, a PCA of 119 East Asian taurine cattle on the basis of SNP genotypes (Figure S5d, Supporting Information) was not sensitive to the use of ARS‐UCD1.2 or Tibetan_v1 as references. This was also found for the calculation of effective population size (*N*e) via a multiple sequentially Markovian coalescent (MSMC),^[^ ^29^ ^]^ which for the Tibetan cattle population has remained relatively stable over the past 10 000 years (Figure S5e, Supporting Information). Therefore, this study confirms that for East Asian taurine cattle, the use of lineage‐specific references can improve variant calling but has a minimal effect on optimizing the evaluation of population genetic structure and effective population size.

### 2.3 SV Detection in QTP Cattle Based on LRS Data

**Figure 2:**
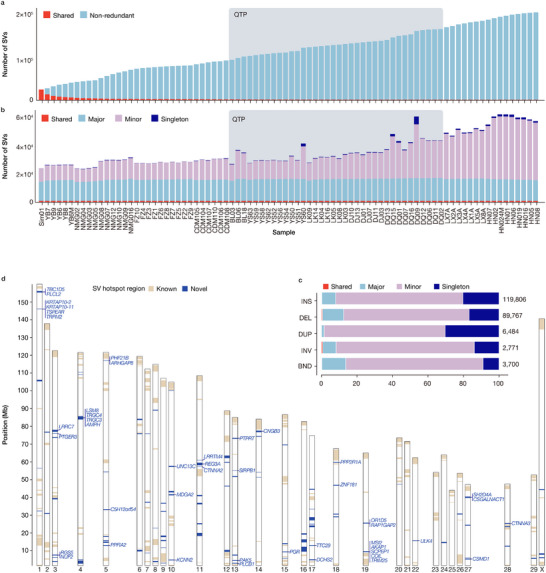
Discovery of structural variations (SVs) in 84 cattle genomes. a) SVs from each sample were merged via a nonredundant strategy starting with Simmental (Sim01) and iteratively adding unique calls from additional samples. The growth rate of the nonredundant set decreases as the number of samples increases. Shared SVs are shown in red color. b) Number of SVs per sample that are classified into shared (identified in all samples), major (in ≥ 42 samples), minor (in > 1 and < 42 samples), and singleton (in only one sample) SVs. c) Proportions of SVs in the four categories defined in (b). d) Chromosomal distribution of SV hotspot regions and 47 SV‐lined genes in newly identified hotspot regions.

We performed LRS of 36 QTP cattle sampled from six locations of Yushu (n = 10), Langkazi (n = 7), Diqing (n = 10), Dingjie (n = 6), and Bailang (n = 3) (Figure 1a; Table S8, Supporting Information). We also generated 28 new LRS genomes and retrieved 20 published LRS genomes of other cattle breeds of Chaidamu (n = 6), Mongolian (n = 10), Yanbian (n = 5), Simmental (n = 1), Hainan (n = 9), Fuzhou (n = 10), and Luxi (n = 7). To keep the coordinates of the genome consistent with previous reports, ARS‐UCD1.2 was used as the reference genome in this study. SVs > 50 bp relative to ARS‐UCD1.2 were detected and retained if they were supported by at least two of the following tools: CuteSV,^[^ ^30^ ^]^ SVIM,^[^ ^31^ ^]^ and Sniffles^[^ ^32^ ^]^ (Figure S6, Supporting Information). On average, 36300 SVs per sample were identified and merged into 222528 nonredundant SVs (**Figure** **2**a,b), including 89767 deletions (DELs), 6484 duplications (DUPs), 119 806 insertions (INSs), 2771 inversions (INVs), and 3700 complex SVs (break‐ends, BNDs) (Figure 2c). Annotation using the ANNOVAR program^[^ ^33^ ^]^ revealed that most of these SVs were located in intergenic and intron regions, with exonic SVs accounting for only 2.2%. Among the 222 528 SVs, 46 028 (20.68%) SVs exhibited homozygous reference genotypes (0/0) across all examined low‐altitude cattle (Yanbian, Simmental, Luxi, Fuzhou, and Hainan), including 16 229 DELs, 27 112 INSs, 1679 DUPs, 458 INVs, and 550 BNDs.

We classified these SVs into four categories: shared (present in all samples), major (present in ≥ 50% of the samples), minor (present in more than one but < 50% of the samples), and singleton (present in only one sample). Among the QTP cattle, Diqing had the most singletons (4.93%), which probably reflects an admixture of East Asian indicine cattle (Figure 1b) or other bovine species (e.g., yak).^[^ ^19^ ^]^ The number of nonredundant SVs initially increased steeply but began to plateau as more samples were added (Figure 2a), suggesting that the 36 cattle captured a substantial proportion of common SVs within the QTP population. The number of nonredundant SVs increases with the addition of taurindicine (Diqing and Luxi) and indicine (Hainan) cattle, which suggests that these are indicine‐specific variants. Although the rate of novel SV discovery gradually slowed with increasing sample size, the continued contribution of non‐redundant SVs—particularly from genetically distinct groups—implies that SV diversity in cattle may not yet be fully saturated. We detected 795 SVs in all samples (Figure 2b), which probably corresponded to singletons or errors in the cattle reference genome.

### 2.4 Characterization of Genomic SVs

Transposable elements (TEs) are important sources of SVs in cattle.^[^ ^34^, ^35^, ^36^ ^]^ A total of 21147 TEs were identified across INS, DEL, and DUP, accounting for 9.50% of all the SVs. The TEs included 20904 retrotransposons (17126 LINEs, 2257 SINEs, and 1521 LTRs) and 243 DNA transposons (Table S9, Supporting Information). The enrichment of LINEs has been reported previously for sheep^[^ ^37^ ^]^ and pigs.^[^ ^38^ ^]^ The SVs displayed sharp peaks at ≈300, 1300, and 8000 bp corresponding to BOV‐A2 (SINE), BTLTR1/BTLTR1B (LTR/ERVK), and L1_BT/LIMA9 (LINE/L1), respectively (Figure S7, Supporting Information).

We identified 259 SV hotspots (see Materials and Methods) with a total size of ≈281 Mb, 157 of which have previously been identified^[^ ^34^, ^39^, ^40^ ^]^ (Figure 2d). Random sampling (1000) and a *Z* test revealed that the SV hotspots are significantly enriched in the TE‐derived SVs, with a major contribution of DEL (*Z* score = 19.67, *P* = 1.81 × 10^−86^) (Table S10, Supporting Information). The same analysis revealed that hotspots are not enriched in conserved element regions found by the genomic evolutionary rate profiling (GERP) (*Z* score = −5.76, *P* = 4.14 × 10^−9^). These findings were consistent with previous reports, which showed that SV hotspots are present mainly in genomic regions rich in genes and high in diversity within populations.^[^ ^34^, ^41^ ^]^ We observed that many newly identified SV hotspot regions, such as those on *B. taurus* autosome 4 (BTA4, *TRGC3* and *TRGC4*), BTA13 (*SIRPB1*), and BTA19 (*TRIM25* and *RAP1GAP2*), are related to immunity (Figure 2d). Notably, the genes *TRGC3* and *TRGC4* on BTA4 belong to the TRG family, which is one of the gene T‐cell receptor subgroups.^[^ ^42^ ^]^

### 2.5 Identification of High‐Altitude‐Adapted SVs

**Figure 3:**
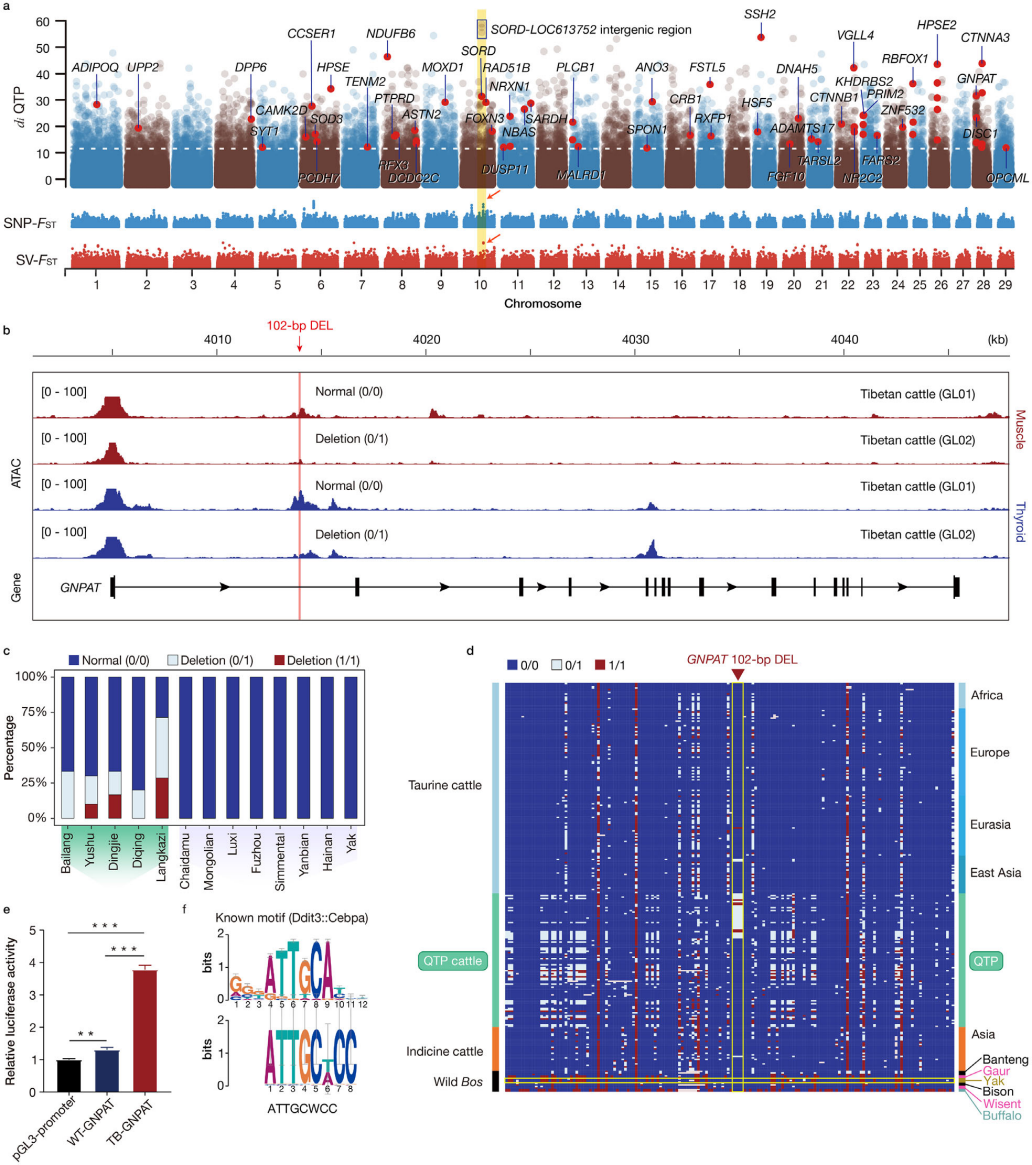
Differentiated structural variations (SVs) associated with the high‐altitude adaptation of Qinghai‐Tibetan Plateau cattle. a) Genome‐wide distribution of *d_i_* for each SV across all autosomes between high‐ and low‐altitude cattle (top panel). The dashed line denotes the significance threshold (*P* < 0.001, corresponding to the *Z* test). The blue block in the Manhattan plot for SNP‐*F*_ST_ and SV‐*F*_ST_ (bottom panel) indicates the consistency of *SORD* region selection for SNPs and SVs. b) The red and blue tracks represent the ATAC‐seq data from muscle and thyroid tissues, respectively, of Tibetan cattle (GL01 and GL02). The pink line shows the 102‐bp DEL (BTA28:4013916‐4014018) in *GNPAT*, located within chromatin accessibility signals. c) Genotype frequency of the 102‐bp DEL in *GNPAT* of the 96 LRS‐based genomes, including data from 84 cattle and 12 yak. d) SNP and SV genotypes in the *GNPAT* region. e) Comparison of the relative luciferase activity between the wild‐type (WT) and Tibetan (TB) cattle haplotypes of *GNPAT*. The bars display the means±SD (n = 3 technical replicates). A two‐sided t‐test was used, where ** indicates *P* < 0.01 and *** indicates *P* < 0.001. f) The top panel shows the known binding motif of the transcription factor Ddit3::Cebpa, while the bottom panel represents the sequence motif identified within the 102‐bp DEL, which matches the binding site of Ddit3::Cebpa. This alignment indicates that the 102‐bp DEL contains the Ddit3::Cebpa binding motif, suggesting that this DEL may affect the function of the regulatory element.

To identify candidate SVs that may play a role in high‐altitude adaptation, we analyzed SVs that have extremely different allele frequencies in high‐altitude QTP cattle relative to low‐altitude cattle using the *d_i_* statistic.^[^ ^43^, ^44^ ^]^ We defined outlier SVs as *d_i_*‐SVs based on the *Z* test (*P* < 0.001) (**Figure** **3**a). Thus, we identified 1692 *d_i_*‐SVs, including 906 INSs, 763 DELs, 16 DUPs, and 10 INVs, involving 601 genes (Table S11, Supporting Information). Functional enrichment analyses of the 601 genes via Kyoto Encyclopedia of Genes and Genomes (KEGG) revealed significant enrichment in pathways (corrected *P* < 0.05) related to cardiac health, metabolic regulation, and neuronal function, including arrhythmogenic right ventricular cardiomyopathy, metabolic pathways, amino sugar and nucleotide sugar metabolism, and the calcium signaling pathway (Figure S8 and Table S12, Supporting Information).

Among the 601 genes, 45 genes have been previously reported with known functions in hypoxic responses and/or as gene candidates associated with physiological traits,^[^ ^45^ ^]^ encompassing 78 SVs (Figure 3a; Table S13, Supporting Information). Several of these SVs are located in genes involved in erythropoiesis and angiogenesis, such as *SSH2*,^[^ ^46^ ^]^ *VGLL4*,^[^ ^47^ ^]^ *PLCB1*,^[^ ^48^ ^]^ *HPSE2*,^[^ ^49^ ^]^ and *HPSE* ^[^ ^50^ ^]^ (Figure 3a). Notably, the 67‐bp INS (BTA19:20937384) in *SSH2* exhibited the highest *d_i_* signal among these genes. As a member of the slingshot phosphatase family, *SSH2* may play an important role in cofilin‐mediated vascular smooth muscle cell migration and neointima formation.^[^ ^46^ ^]^ *HPSE* and *PLCB1* are associated with the adaptation of Tibetan^[^ ^51^ ^]^ and alpine Merino sheep^[^ ^52^ ^]^ to high‐altitude hypoxia, respectively. In addition, we detected an 8493‐bp INS (BTA22:13818542) in *CTNNB1*, with an allelic difference of 0.211 (SV‐*F*_ST_) between QTP and low‐altitude cattle. *CTNNB1* knockdown inhibited hypoxia‐regulated gene expression in vitro and reduced the protein level of hypoxia‐inducible factor‐1α (HIF‐1α).^[^ ^53^ ^]^

We further detected several important candidate SV‐related genes associated with lipid metabolism and mitochondrial metabolism, which are crucial for oxygen homeostasis.^[^ ^54^ ^]^ The strongest signals were observed on BTA10, including four consecutive SVs of 74‐bp DEL (BTA10:65366980‐65367054), 71‐bp INS (BTA10:65418831), 601‐bp INS (BTA10:65422143), and 1056‐bp DEL (BTA10:65424402‐65425458) in the intergenic and intronic regions of the sorbitol dehydrogenase (*SORD*) gene (Figure 3a; Figure S9, Supporting Information). The allele frequencies of these SVs were as high as 35.7 to 83.3% in QTP cattle (Figure S9, Supporting Information). These SVs shared the same selection signatures of their linked SNPs (Figure 3a). *SORD* is the second enzyme of the polyol pathway. Low expression of *SORD* is expected to attenuate the increase in the cytosolic NADH/NAD^+^ ratio and increase glycolysis.^[^ ^55^ ^]^ Another highly *di*‐SV is an 86‐bp DEL in the intron of *NDUFB6* (BTA8:11510584‐11510670). This is a crucial component of respiratory complex I, which, together with complex III generates most of the mitochondrial ROS.^[^ ^56^, ^57^ ^]^ A third top signal is a 52‐bp DEL (BTA11:104554345‐104554397) within the intron of *SARDH*. This gene encodes an enzyme localized to the mitochondrial matrix and plays an important role in promoting energy balance in the context of nutrient stress through the CREB/CRTC pathway.^[^ ^58^ ^]^ We also detected a 65‐bp INS (BTA1:80427709) located upstream of *ADIPOQ*, which is involved in adipose metabolism, body homeostasis, and cardiovascular protection.^[^ ^59^ ^]^

To explore the potential regulatory roles of the 1692 *d_i_*‐SVs under selection, we integrated ATAC‐seq data from six key tissues (adipose, liver, heart, lung, muscle, and thyroid) from high‐ and low‐altitude cattle to identify chromatin accessible regions (CARs) (Table S14, Supporting Information). By intersecting with CARs, we identified 59 *d_i_*‐SVs (3.5%) and 75 SV‐associated genes (Table S15, Supporting Information). Notably, three genes (*GNPAT*, *CAT*, and *ACOT2*) were significantly enriched in the GO pathway of the peroxisomal matrix. *GNPAT* encodes glyceronephosphate O‐acyltransferase, which is located in the peroxisomal membrane and catalyzes the first step of ether lipid synthesis involved in the decomposition of hydrogen peroxide and the maintenance of reactive oxygen species homeostasis.^[^ ^60^, ^61^ ^]^ We identified a 102‐bp DEL in the first intron of *GNPAT* (BTA28:4013916‐4014018) (Figure 3a,b), which had the highest signal among these three SVs. This 102‐bp DEL was found in 13 out of the 36 LRS‐based (36%, Figure 3c) and 36 out of the 112 SRS‐based genomes (32%, Figure 3d; Figure S10, Supporting Information) of QTP cattle, with a very low detection rate in other cattle breeds. A previous study indicated that peroxisomal genes play crucial roles under hypoxic conditions.^[^ ^54^ ^]^ ATAC‐seq data from different tissues of QTP cattle revealed that the 102‐bp DEL falls within an open chromatin region. Chromatin accessibility at this locus was reduced in the heterozygous GL02 individual (genotype 0/1) compared to the wild‐type GL01 (genotype 0/0) (Figure 3b; Figure S11, Supporting Information).

Compared with the normal taurine haplotype, the QTP cattle‐specific haplotype carrying the 102‐bp DEL exhibited greater activity in a luciferase assay (*P* < 0.001) (Figure 3e). This finding indicates that the DEL may disrupts the structure of the open chromatin and subsequently affect *GNPAT* expression. Within the 102‐bp sequence, we identified a putative CCAAT/enhancer‐binding protein (C/EBP)‐binding motif (Ddit3::Cebpa), which controls lineage‐specific gene expression and acts as a master regulator of the terminal differentiation of several cell types^[^ ^62^ ^]^ (Figure 3f). Notably, the *GNPAT* gene region was found to be introgressed from yak to QTP cattle.^[^ ^19^ ^]^ However, yak carried this 102‐bp fragment (Figure 3d), suggesting that this 102‐bp DEL was not derived from yak introgression.

### 2.6 Yak Introgression

**Figure 4:**
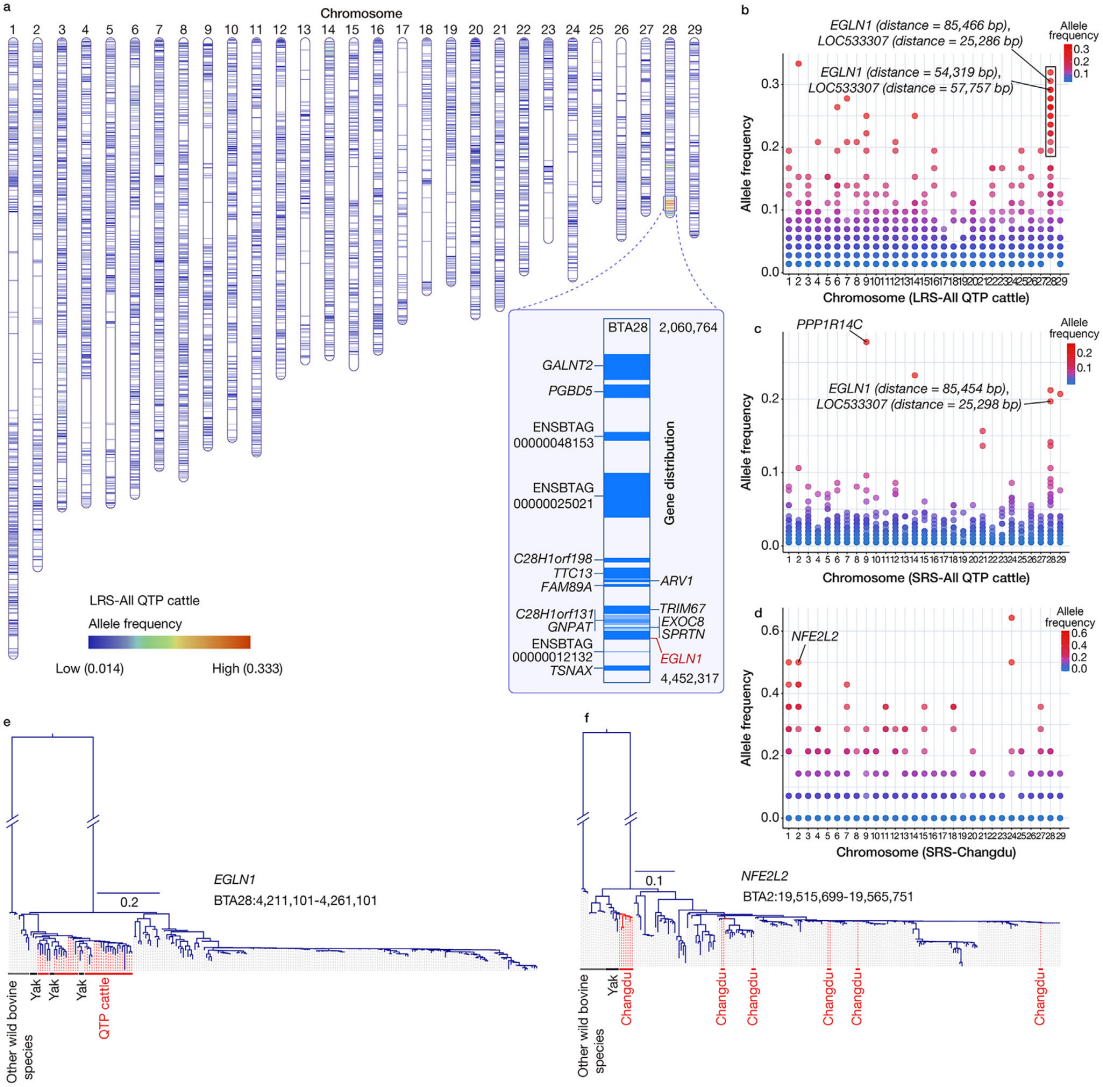
Genome‐wide introgression from yak into Qinghai‐Tibetan Plateau (QTP) cattle. a) The distribution of yak introgressed fragments in the genome based on LRS‐SV results, as well as the SV hotspot region on BTA28. b–d), Allele frequencies of the SVs resulting from yak introgression b) in the LRS‐based genomes of all QTP cattle (LRS‐All QTP cattle); c) in the SRS‐based genomes of all QTP cattle (SRS‐All QTP cattle); and d) in the SRS‐based genomes of Changdu cattle (SRS‐Changdu). e) Phylogenetic analysis of the SNPs in the introgressed *EGLN1* gene. f) Phylogenetic analysis of the SNPs in the introgressed *NFE2L2* gene.

Previous studies have reported that historic yak introgression events have contributed to the rapid adaptation of QTP cattle.^[^ ^13^, ^14^, ^63^ ^]^ We used LRS‐based genomes of 12 yak to identify the SVs that may have resulted from the introgression of yak into QTP cattle (Table S8, Supporting Information). We then searched for SVs that were shared by yak genomes but not present in other cattle genomes. Using LRS‐based genomes, 7293 autosomal SVs possibly derived from yak introgression were identified in QTP cattle, totaling 3.38 Mb (**Figure** **4**a). To further validate these regions, we constructed a neighbour‐joining (NJ) tree using SNPs from 50‐kb flanking regions of the SVs in QTP cattle and yak, tracing the haplotype origins. In this way, we identified 5642 SVs in QTP cattle as probable yak introgressions (Table S17, Supporting Information). We found an introgressed SV hotspot region on BTA28, including two SVs (1324‐bp DEL in BTA28:4236113‐4237437 and 570‐bp INS in BTA28:4204966) located upstream of the well‐known hypoxia‐inducing gene *EGLN1*.^[^ ^64^ ^]^ The two SVs had high frequencies in QTP cattle (Figure 4a‐c,e). Another high‐signal SV was a 57‐bp DEL (BTA9:87336841‐87336898) located in the first intron of *PPP1R14C*, a gene associated with angiogenesis.^[^ ^65^ ^]^

To expand the population size, the SVs were further genotyped using 293 SRS‐based genomes, from 112 QTP cattle, 169 cattle from various global populations, and 12 individuals from other bovine species with high‐coverage genome sequences (Table S16, Supporting Information). Applying the same identification criteria used for the LRS data, we detected 2334 SVs possibly derived from yak introgression (Table S18, Supporting Information), of which 971 showed overlap with the LRS SVs. Consistent with a recent study using SNP data,^[^ ^19^ ^]^ our results indicate that not all QTP cattle populations have the same yak introgression signals. For example, the top candidate regions in the Changdu cattle population differ from those in other QTP cattle populations (Figure 4d). Interestingly, an introgressed region on BTA2, including a 52‐bp DEL in the intron of *NFE2L2* (BTA2:19540699‐19540751), has a high frequency in the Changdu population and is absent in other cattle (Figure 4f). *NFE2L2* (NFE2‐like bZIP transcription factor 2, also known as *NRF2*) plays a key role in the response to oxidative stress and induces the upregulation of glucose transporter 1 (*GLUT1*), which allows increased glucose uptake and HIF‐1‐driven augmentation of glycolysis.^[^ ^66^ ^]^

### 2.7 A 2‐Mb Heterozygous Inversion Related to the Gray Coat Phenotype of QTP Cattle

**Figure 5:**
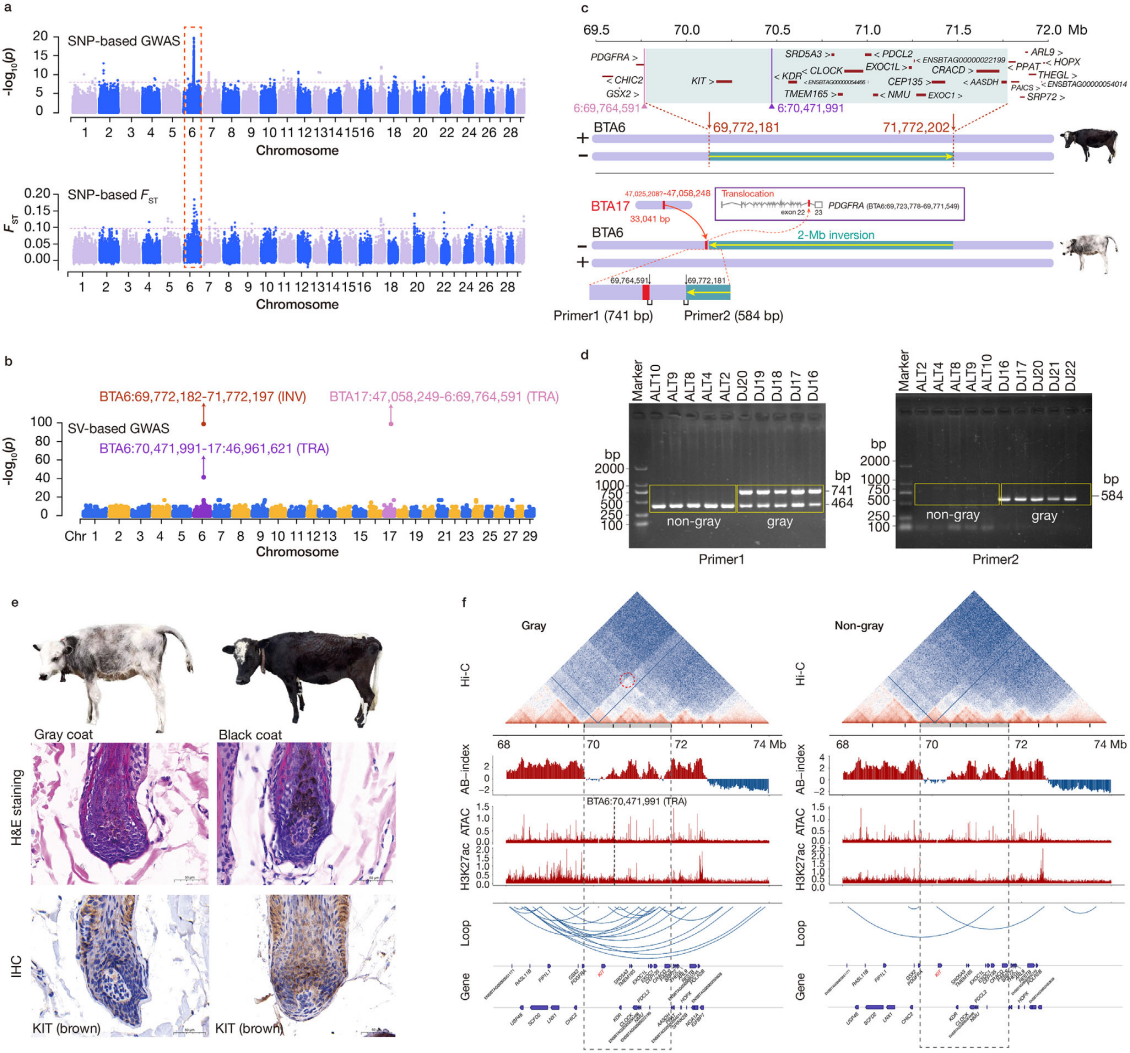
A 2‐Mb heterozygous inversion related to the gray coat phenotype of Qinghai‐Tibetan Plateau (QTP) cattle. a) Genome‐wide Manhattan plots generated via GWAS and *F*_ST_ based on SNPs for the gray coat phenotype. b) Genome‐wide Manhattan plot using the GWAS based on SVs for gray coats. c) Rearrangement of the 2‐Mb INV in non‐gray (top) and gray (bottom) QTP cattle. This region displays the genes located on BTA6 between positions 69.50‐72.00 Mb in the *B. taurus* reference genome (ARS‐UCD1.2) from the Ensembl database, with the blue shaded area indicating the specific location of the inversion at BTA6:69772181‐71772202. In addition to the 2‐Mb inversion on BTA6, we also identified two translocations (pink and purple lines), one of which involves a fragment from BTA17 that is translocated into intron 22 of *PDGFRA*. d) PCR validation of the 2‐Mb INV. We designed two pairs of PCR primers at the insertion site of the translocation (Primer1, BTA6:69764591) and the starting position of the inversion (Primer2, BTA6:69772181) and validated them in gray and non‐gray cattle. e) Skin sections of gray and black cattle stained with hematoxylin and eosin (H&E) and subjected to immunohistochemistry (IHC), respectively. For the IHC analysis, a rabbit antibody against KIT was used, with the brown color indicating positive staining and the blue color representing the cell nuclei. Scale bars, 50 µm. f) Chromatin architecture and regulatory landscape in the gray and non‐gray cattle genomic regions flanking the 2‐Mb INV region on BTA6. The top section represents a Hi‐C contact map, which visualizes chromatin interactions, with red and blue colors indicating high interaction densities. The grey shaded area encompasses four consecutive TADs in the cattle genome, with a highlighted region (red dashed circle) indicating strong chromatin interactions in gray cattle. The AB index plot, where red bars represent active (A) compartments and blue bars denote inactive (B) compartments. The ATAC and H3K27ac tracks show regulatory activity in this region. Loops derived from Hi‐C data highlight chromatin interactions across this region. The bottom panel shows an annotation map of genes located on BTA6 between positions 68–74 Mb in the ARS‐UCD1.2 genome from the Ensembl database.

We further examined QTP‐specific SVs and found a 2‐Mb heterozygous INV and two translocations (TRAs) related to the gray coat phenotype in cattle. The gray phenotype of mammals occurs when at birth the hairs lose their pigment and turn white. This is observed in gray horses,^[^ ^67^ ^]^ but also with a low frequency in QTP cattle. To confirm the genomic candidate region of the gray coat phenotype, SRS data from 23 gray cattle and 258 non‐gray cattle were used for SNP‐based GWAS (SNP‐GWAS) and SNP‐based *F*_ST_ (SNP‐*F*_ST_) analyses (**Figure** **5**a). Both SNP‐GWAS, which uses the genome‐wide efficient mixed model association (GEMMA) program with a mixed linear model, and SNP‐*F*_ST_ confirmed a strong signal on BTA6. The GWAS signal spans 2.99 Mb (BTA6:69765842‐72752916; *P* < 1 × 10^−8^). This region harbors 23 non‐LOC genes and 13 LOC genes, including *KIT* (Figure S12, Supporting Information). The signals of the SNP‐*F*_ST_ contrasting 22 gray and 29 non‐gray QTP cattle (BTA6:69750001‐72725001, *P* < 0.001) overlapped with the SNP‐GWAS signals (Figure 5a). However, no functional SNP or InDel was found. We then performed a whole‐genome SV‐based GWAS (SV‐GWAS) (Figure 5b) and SV‐based *F*_ST_ (SV‐*F*_ST_) analysis (Figure S13, Supporting Information), and confirmed the highest signal of the 2‐Mb heterozygous INV (BTA6:69772181‐71772202), together with two TRAs in BTA17 (47058249) and BTA6 (70471991) (Figure 5b), overlapping with the region detected by SNPs for the gray coat color phenotype in cattle.

The INV was confirmed by analysis of discordant and split reads at the junctions in gray cattle compared with those at the junctions in non‐gray cattle (Figure S14, Supporting Information). We further confirmed the presence of this 2‐Mb heterozygous INV via the program npInv,^[^ ^68^ ^]^ an accurate tool for detecting and genotyping inversions using multiple alignment long reads. This INV was probably caused by error‐prone nonhomologous end joining (NHEJ) events and contains 20 coding genes, including *KIT* (Figure 5c). There was no disrupted gene present at the INV boundary; however, a translocation fragment of ≈33 kb from BTA17 was found 7590 bp upstream of the INV proximal breakpoint (Figure 5b,c, Supporting Information). This fragment was inserted into the 22nd intron of *PDGFRA*. We confirmed the INV and TRAs by PCR in 23 other gray cattle (Figure 5d; Figure S15, Supporting Information).

To further investigate the possible origin of this INV in QTP cattle, we conducted a comprehensive haplotype phylogenetic analysis using sequences from the INV region (BTA6:69722181‐71822202). The results indicate that the INV‐carrying haplotypes show strong phylogenetic clustering with South Asian indicine cattle (*Bos indicus*), while the alternative haplotype primarily groups with East Asian taurine cattle (*Bos taurus*) (Figure S16, Supporting Information). This suggests that the inversion haplotype segment was likely originated from South Asian indicine cattle, consistent with the presence of South Asian indicine ancestry components in QTP cattle populations as previously reported.^[^ ^19^ ^]^

Due to the well‐established role of *KIT* in determining white‐spotting phenotypes in several species, this gene is an obvious candidate for the gray coat phenotype in QTP cattle. *KIT* is expressed in several tissues, particularly in mast cells and melanocytes.^[^ ^69^ ^]^ Using H&E staining and immunohistochemistry of skin samples, we observed a more intense presence of melanin and more pronounced KIT signals around the hair follicles of black cattle than in those of gray cattle (Figure 5e). Hi‐C data from gray cattle and non‐gray cattle indicated that the breakpoints of this INV were located at topologically associated domains (TAD) boundaries (Figure 5f; Table S19, Supporting Information). The presence of the INV led to stronger interactions across the TAD boundaries and stronger H3K27ac signals upstream of the inversion in gray cattle. Evolutionary conservation was confirmed through cross‐species alignment (LiftOver; BTA6:68‐70 Mb to GRCm39), showing mice *KIT* enhancer sequences are conserved in cattle (Figure S17a, Supporting Information), with these enhancers known to reside within *KIT* TAD in mice melanocytes.^[^ ^69^ ^]^ Furthermore, the TRA at BTA6:70471991 in gray cattle precisely localizes to the CTCF‐defined TAD border between *KIT* and *KDR* (Figure 5f; Figure S17b, Supporting Information), while deletion of the *Kit*‐*Kdr* TAD boundary in mice results in a lighter coat color phenotype.^[^ ^69^ ^]^ Together, these findings suggest that the INV and associated TRA events may alter *KIT* expression through TAD boundary disruption and consequent enhancer dysregulation, contributing to the gray coat phenotype in QTP cattle. We then examined the linkage disequilibrium (LD) level between the SNPs positioned within or adjacent to the 2‐Mb heterozygous INV (Figures S18 and S19, Supporting Information). The LD levels between distant SNPs are generally higher in gray cattle than in non‐gray cattle, indicating a suppression of recombination by the large inversion.

Previous studies have established the cell‐specific expression pattern of the *Kit* gene in mice, leading us to hypothesize that the effects of INV might exhibit cell‐type specificity (e.g., melanocytes). However, current bulk‐level Hi‐C interaction data lack resolution to discern such cellular heterogeneity. Future investigations employing single‐cell Hi‐C or other high‐resolution chromatin conformation capture methods could help verify these possibilities and elucidate cell‐type‐specific regulatory mechanisms.

## 3 Discussion

In this study, we generated a high‐quality assembly of Tibetan taurine cattle (Tibetan_v1) using a combination of PacBio HiFi and Hi‐C technologies. Tibetan_v1 has quality metrics comparable to or even exceeding those of other currently available cattle reference genomes. It may serve as a reference genome serve as an important data resource for further analysis of Asian cattle breeds, particularly for studies of the bovine pangenome.^[^ ^70^ ^]^ Bovine autosomes are acrocentric,^[^ ^71^ ^]^ and a complete bovine assembly is close to “centromere‐to‐telomere”.^[^ ^72^ ^]^ We identified 20 autosomes with telomeric repeats in the Tibetan_v1 assembly.^[^ ^72^ ^]^ In the application of population genetics, the use of a population‐specific reference genome (Tibetan_v1) instead of ARS‐UCD1.2 more accurately distinguishes East Asian cattle, detects rare low‐frequency variants, and allows the conversion of multiallelic into biallelic variants. The multiallelic variants covered by the European bovine reference genome in QTP cattle are mainly restricted to highly polymorphic immune‐related genes (*BoLA*, *BoLA‐NC1*, etc.).^[^ ^73^, ^74^ ^]^ Our results suggest that sequences with high nucleotide diversity will benefit from population‐specific reference genomes. This observation aligns with findings in other species, such as Tibetan goat, where population‐specific reference genomes have been shown to improve the detection of novel selective regions.^[^ ^75^, ^76^ ^]^

Whole‐genome scanning of nucleotide variations has been widely used to identify candidate genes for hypoxia adaptation in high‐altitude species.^[^ ^19^ ^]^ In this study, we focused on the role of SVs in high‐altitude adaptation, which involves both polygenic inheritance and gene introgression. We generated the largest dataset of QTP cattle SVs to date and identified SVs that are highly differentiated between high‐ and low‐altitude cattle populations, potentially linked to high‐altitude adaptation. Increasing oxygen delivery and reducing oxygen consumption are the two primary responses to hypoxia; the former relies on the improvement of blood and vascular conditions, and the latter mainly refers to glucose and lipid metabolism.^[^ ^77^ ^]^ We identified SVs associated with high‐altitude adaptation in QTP cattle, primarily overlapping with genes related to erythropoiesis and angiogenesis (*SSH2*, *VGLL4*, *PLCB1*, *HPSE2*, and *HPSE*) and energy metabolism (*SORD*, *NDUFB6*, *SARDH*, and *ADIPOQ*). Similar adaptive pathways (e.g., hypoxia response and energy metabolism) were previously observed in yak,^[^ ^1^, ^63^, ^78^ ^]^ though involving distinct genes, suggesting convergent mechanisms for high‐altitude survival between QTP cattle and native yak.

The selection signal around the *SORD* gene was highly consistent in both the SNPs and the SVs between the high‐ and low‐altitude cattle populations, indicating its potential crucial role in high‐altitude adaptation. A previous study on ischemia/reperfusion‐induced injury of the rat heart revealed that *SORD* (also called *SDH*) affects the induction of HIF‐1α by regulating the level of NAD^+.[^ ^55^ ^]^ HIF‐1α is a subunit of a transcription factor called hypoxia‐inducible factor 1.^[^ ^79^ ^]^ The physiological function of HIFs is to promote the adaptation of cells to low oxygen by inducing neovascularization and glycolysis.^[^ ^80^ ^]^ Other high‐signal SV‐associated genes, such as *CTNNB1^[^* ^53^ *^]^* and *HPSE*,^[^ ^81^ ^]^ may also help the high‐altitude adaptation of QTP cattle through the HIF pathway. Notably, some strongly selected SVs (e.g., *SSH2*) were located outside ATAC‐seq predicted open chromatin regions. This observation suggests two non‐exclusive possibilities: 1) The regulatory effects of adaptive SVs may operate through mechanisms independent of canonical chromatin accessibility; 2) Tissue‐specific regulatory landscapes‐while our ATAC‐seq covered six major tissues (heart, liver, lung, muscle, adipose and thyroid), key regulatory elements for certain genes might be active in other cell types. This highlights the importance. This highlights the necessity for future studies to integrate broader tissue sampling, single‐cell multi‐omics profiling, and functional validation to comprehensively resolve the regulatory mechanisms underlying high‐altitude adaptation. Additionally, we identified *d_i_*‐SVs in three peroxisomal genes (*GNPAT*, *CAT*, and *ACOT2*). We detected a 102‐bp DEL in the first intron of *GNPAT*, which is located within a predicted chromatin open region. The results from the luciferase reporter assay showed that the fragment containing the 102‐bp DEL significantly increased luciferase expression in mouse 293T cells, suggesting that the DEL sequence may block the function of a regulatory element.^[^ ^82^ ^]^ In Tibetans, *GNPAT* has also been identified as a key gene involved in the response to UV treatment through the promotion of melanin synthesis.^[^ ^83^ ^]^ *CAT* encodes a catalase that plays a key role in protecting cells and dismutates H_2_O_2_ to water and oxygen in response to oxidative stress. Combined with previous study,^[^ ^54^ ^]^ it suggests that peroxisomal genes may play crucial roles under hypoxic conditions in QTP cattle.

BTA28 contains relatively many yak‐introgressed regions, which is consistent with previous SNP‐based analyses.^[^ ^19^ ^]^ Notably, this region includes hypoxia genes such as *EGLN1*, *EXOC8*, *GNPAT*, *SPRTN*, *TSNAX*, *FAM89A*, *TRIM67*, and *ARV1*.^[^ ^54^, ^83^, ^84^ ^]^ Interestingly, the same genomic region has also been reported in high‐altitude species such as the zoker (*Eospalax baileyi*)^[^ ^85^ ^]^ and Tibetan humans.^[^ ^86^ ^]^ SNPs revealed that the proportions of yak ancestry ranged from 0.64% to 3.26% in the QTP cattle genomes, among which Nangqian, Changdu, and Yushu were the highest, followed by Diqing, Zhangmu, Dingjie, and Shigatse taurine cattle.^[^ ^19^ ^]^ In this study, we identified distinct hotspots of SV introgression across different QTP populations, indicating various patterns of yak introgression. For example, in the Changdu population, the highest introgression region signal was found on BTA2, which includes the *NFE2L2* gene, which is involved in the oxidative stress response.^[^ ^87^ ^]^ Changdu cattle inhabit an agro‐pastoral zone where interbreeding with yak is common. Our results indicate that not all QTP cattle populations have the same yak introgression signals or similar adaptive mechanisms, which may reflect the size of the QTP with geographical barriers. This has also influenced human variation. The top candidate genes that carry adaptive genetic variation previously found in Tibetan humans, such as *EPAS1* and *EGLN1*, do not have strong positive selection signals in Deng people, who also live on the Plateau and have selection signals in *KLHL12* and other genes.^[^ ^88^ ^]^

Chromosomal INVs may affect recombination and chromosome structure. However, INVs can be challenging to detect, so the prevalence and hence the significance of INVs segregating within a species are often overlooked. LRS data for gray cattle revealed a 2‐Mb heterozygous INV that distinguished phenotypically gray cattle from non‐gray cattle. These INVs may very well be favored by natural selection because they suppress recombination between coadapted genes,^[^ ^89^ ^]^ where the gray coat phenotype contributes to adaptation to high‐altitude environments. Previous studies in humans and other mammals have shown that large INVs modify gene expression via two major mechanisms: i) directly disrupting regulatory elements adjacent to breakpoints and ii) rearranging regulatory elements positioned within or near the inverted region.^[^ ^89^ ^]^ At the same time, we observed that the 2‐Mb INV was accompanied by two TARs, a phenomenon that appears to be common in cytogenetically detected INVs.^[^ ^90^ ^]^ At the INV breakpoints, complexity and imbalances are often observed, which may result from error‐prone repair of processed double‐strand breaks.^[^ ^91^ ^]^ Inhibitory recombination of inverted heterozygotes has been reported in several species, such as flat‐fruit peach^[^ ^92^ ^]^ and deer mice.^[^ ^93^ ^]^ A light coat color may contribute to adaptation to high‐latitude or tropical/subtropical environments by reflecting a relatively large proportion of incident solar radiation.^[^ ^94^ ^]^ We propose that the gray coat phenotype in cattle arises through structural rearrangement of regulatory elements by this INV, or alternatively, through potential disruption of the *KIT*‐*KDR* TAD by the TRA, leading to dysregulated *KIT* expression.

GWAS based on SNPs constitute a powerful experimental strategy for identifying genetic variations underlying animal traits.^[^ ^95^, ^96^ ^]^ However, causative genomic variations often involve long stretches of DNA, such as the 2‐Mb heterozygous INV identified in this study, and are not detected by genome‐wide SNP genotyping. Our study suggests that LRS analysis can identify important SVs that SRS data cannot detect, providing high‐confidence candidate genes and variants for subsequent functional studies.

## 4 Conclusion

We generated a high‐quality genome assembly for Tibetan cattle and presented a comprehensive catalog of SV variations. We evaluated the important role of the population‐specific reference genome in detecting rare and low‐frequency variations. Leveraging the advantages of population‐scale long‐read sequencing, we identified genes and related SVs associated with high‐altitude adaptation and coat phenotypes, offering new insights into the plateau adaptability of cattle to the QTP.

## 5 Experimental Section

### Ethics Statement

This study was approved by the Institutional Animal Care and Use Committee of Northwest A&F University (FAPWCNWAFU, Protocol no. NWAFAC 1008).

### Samples Collected for Tibetan Cattle Genome Assembly

Whole blood from the Dingjie bull DJ12 was collected to construct the Tibetan cattle genome assembly. The bull was located in Dingjie County, Shigatse City, Xizang Autonomous Region, China. Genomic DNA was extracted from the tissues of the animals using the phenol/chloroform method. The resulting DNA was sequenced using the Pacific Biosciences (PacBio) Sequel II platform at Novogene, yielding a total of 102 Gbp, corresponding to a genomic coverage of ≈39×. In addition to long reads, the same animal was resequenced using Illumina HiSeq X Ten paired‐end short‐read sequencing, yielding 376 Gbp with an average insert size of 350 bp, corresponding to a genomic coverage of ≈145×.

### In Situ Hi‐C Library Preparation

In this study, one library was generated from the blood of the Dingjie bull DJ12 and six in situ Hi‐C libraries from the skin tissue of gray and non‐gray cattle. Hi‐C libraries were constructed according to previous studies.^[^ ^97^ ^]^ Briefly, the samples were crosslinked with 1% formaldehyde for 10 min at room temperature and quenched with 0.125 M glycine for 5 min. The crosslinked cells were subsequently lysed. Endogenous nuclease was inactivated with 0.3% SDS, and chromatin DNA was digested with 100 U of MboI (NEB), marked with biotin‐14‐dCTP (Invitrogen), and then ligated with 50 U of T4 DNA ligase (NEB). After the crosslinking was reversed, the ligated DNA was extracted with a QIAamp DNA Mini Kit (Qiagen) according to the manufacturer's instructions. The purified DNA was sheared into 300‐ to 500‐bp fragments and subjected to further blunt‐end repair, the addition of A‐tails and adaptors, followed by purification using biotin‐streptavidin‐mediated pull‐down and PCR amplification. Finally, the Hi‐C libraries were quantified and sequenced on the MGI‐seq platform (BGI, China).

### Whole‐Genome Nanopore LRS Samples

Blood or ear tissue samples were collected from 36 cattle in five high‐altitude regions (above 3000 m) in the Xizang Autonomous Region, Qinghai, and Yunnan Province, China. Low‐altitude cattle were collected from Qinghai, Jilin, and Shandong Province, all below 3000 m above sea level, for a total of 28 blood or ear tissue samples. The sequencing libraries were prepared using an SQK‐LSK109 ligation kit (Oxford Nanopore Technologies; Oxford, UK). Sequencing was performed using PromethION flow cells (R9.4). Base calling was performed using Guppy (v.5.1.13). In addition, 20 published LRS datasets were downloaded from low‐altitude cattle for joint analysis. LRS data were downloaded from six domestic yak and six wild yak from the NCBI to identify SVs. All the samples used in this study are listed in Table S8 (Supporting Information).

### Samples Collected for Whole‐genome Illumina SRS

The Illumina SRS data of 281 cattle from six geographic regions (Africa, Europe, Eurasia, Northeast Asia, South China, and South Asia) as well as six wild bovine species (n = 12) were generated in our study or retrieved from the sequence read archive of the National Center for Biotechnology Information (NCBI, Table S16, Supporting Information).

### Genome Assembly and Quality Assessment

Hifiasm (v.0.16.1‐r375) was used to generate the assembly from HiFi CCS reads with default parameters.^[^ ^25^ ^]^ Hifiasm yields one primary contig assembly and a pair of partially phased contig assemblies. The primary contigs were scaffolded to chromosomes using clean Hi‐C reads with Juicer (v.1.5)^[^ ^98^ ^]^ and 3D‐DNA (v.201008).^[^ ^99^ ^]^ The assembly was manually reviewed and adjusted using Juicebox Assembly Tools.^[^ ^100^ ^]^ Genome completeness was assessed using BUSCO (v.4.0)^[^ ^101^ ^]^ with the Mammalia_odb10 database. Merqury (v.1.0)^[^ ^27^ ^]^ was used to estimate the quality value (QV) by employing high‐depth Illumina SRS data from the same individual whose genome was assembled. Mash^[^ ^26^ ^]^ was used to estimate the distance between the assembled genome of the Tibetan cattle genome and other genomes.

### Genome Annotation

Repetitive elements in Tibetan_v1 were identified by their matches to Repbase (v.20140131) using RepeatMasker (v.4.0.5) (http://www.repeatmasker.org). To identify protein‐coding genes in Tibetan_v1, Liftoff (v.1.5.2)^[^ ^102^ ^]^ and GffRead (v.0.12.1)^[^ ^103^ ^]^ were used to generate gene annotations from ARS‐UCD1.2. The annotation used for the transfer was NCBI GCF_0 022 63795.1_ARS‐UCD1.2_genomic.gff.

### Variant Calling Evaluation

To assess the variant calling performance of the different references, the SRS data of 27 Dingjie samples were aligned to each of the two assemblies (ARS‐UCD1.2 and Tibetan_v1) using BWA‐MEM (v.0.7.13‐r1126) with default parameters, and the duplicated reads were removed using Picard Tools (http://broadinstitute.github.io/picard). The Genome Analysis Toolkit (GATK, v.3.8‐1‐0‐gf15c1c3ef) was used to detect SNPs and InDels. The variations were called using the “HaplotypeCaller”, “GenotypeGVCFs”, and “SelectVariants” of GATK. After SNP or InDel calling, “VariantFiltration” was used to discard sequencing and alignment artifacts from the SNPs with the parameters ′QD < 2.0, FS > 60.0, MQ < 40.0, MQRankSum < −12.5, ReadPosRankSum < −8.0, and SOR > 3.0″ and based on a mean sequencing depth of variants (all individuals) of “< 1/3×and > 3×.” To identify the biallelic SNPs in Tibetan_v1 that appeared to be multiallelic under ARS‐UCD1.2, first the biallelic SNPs were extracted from Tibetan_v1 and the multiallelic SNPs from ARS‐UCD1.2 in 27 Dingjie cattle. Then, using the chain file created between Tibetan_v1 and ARS‐UCD1.2, LiftOver tools were employed for coordinate conversion.

### Population Structure Analysis and Estimation of the Effective Population Size

To examine the fine‐scale population structure revealed by genetic variants from different assemblies (ARS‐UCD1.2 and Tibetan_v1), PCA of cattle populations was performed in the QTP region and northern China. PCA was conducted using the smartPCA program in EIGENSOFT (v.5.0).^[^ ^104^ ^]^ A neighbor‐joining (NJ) tree was constructed using the matrix of pairwise genetic distances calculated by PLINK (v.1.9).^[^ ^105^ ^]^ The unrooted NJ tree was then visualized via MEGA (v.5.0)^[^ ^106^ ^]^ and FigTree (v.1.4.3) (http://tree.bio.ed.ac.uk/software/fgtree/). A NeighborNet network was constructed using Reynold's distances between populations using SplitsTree App (v.6.3.32).^[^ ^107^ ^]^

For the genetic variants called from different reference assemblies (ARS‐UCD1.2 and Tibetan_v1), MSMC2 analysis was applied to estimate the effective population size (*N*e) of the Tibetan cattle, Anxi cattle, and Angus populations over time. This method was applied to all groups with two deep‐coverage (>14 ×) individuals per group. The samples (coverage) used in this analysis were as follows: Anxi, O718844 (32.72 ×) and V347862 (33.36 ×); Angus, SRR1365144 (16.27 ×) and SRR1425124 (16.44 ×); and Tibetan cattle, Xizang22 (26.28 ×) and Xizang7 (24.56 ×). For the calculation of the effective population size, the parameters of MSMC2 were set to ′msmc2 ‐t 10 ‐p 1*2+25*1+1*2 ‐I 0, 1, 2, 3″ and ′msmc2 ‐t 10 ‐p 1*2+25*1+1*2 ‐I 4, 5, 6, 7″. For the calculation of population separation, the parameters of MSMC2 were set to ′msmc2 ‐t 8 ‐P 0, 0, 0, 0, 1, 1, 1, 1 ‐s ‐p 1*2+25*1+1*2″. For effective population size inference, two individuals (4 phased haplotypes) from each group were used. A time scale for generation time of *g* = 6 and a mutation rate per generation of µg = 1.26 × 10^−8^ were used.

### SV Discovery and Genotyping

LRS data from 84 samples were aligned to ARS_UCD1.2. Mapping was performed using NGMLR (v.0.2.7)^[^ ^32^ ^]^ with default parameters. SV calling was performed using CuteSV (v.2.0.3),^[^ ^30^ ^]^ SVIM (v.1.4.0),^[^ ^31^ ^]^ and Sniffles (v.2.2).^[^ ^32^ ^]^ Three minimum supporting reads were needed for each SV. The minimum length of the SVs was at least 50 bp. Specifically, CuteSV was run with the parameters ′–max_cluster_bias_INS 100 –diff_ratio_merging_INS 0.3 –max_cluster_bias_DEL 100 –diff_ratio_merging_DEL 0.3 –min_support 3 –min_size 50 –genotype –report_readid –sample.″ The following parameters were set for SVIM: ′–min_sv_size 50 –min_mapq 20 –minimum_depth 3 –insertion_sequences –sequence_alleles –read_names –sample.″ Sniffles was run with the parameters “–minsupport 3 –output‐rnames” to remove positions marked as IMPRECISE for INFO or as UNRESOLVED for FILTER. SURVIVOR (v.1.0.7)^[^ ^108^ ^]^ was used to merge the SVs supported by two or three calling methods, with a maximum allowed pairwise distance of 500 bp between breakpoints. Individuals were genotyped using the force calling function of Sniffles with the –genotype‐vcf option. Multisample VCF files were merged and deduplicated using BCFtools (v.1.17)^[^ ^109^ ^]^ and the “collapse” module in Truvari software.^[^ ^110^ ^]^ After filtering out unreliable genotypes (all 0/0), a total of 222 528 SVs were obtained.

These 222 528 SVs were genotyped in 281 cattle and 12 individuals from other bovine species with available SRS data. Reads were mapped to ARS‐UCD1.2 using BWA‐MEM (v.0.7.13‐r1126) with default parameters. Paragraph (v.2.4a)^[^ ^111^ ^]^ was used to genotype the SVs from the SRS data. BCFtools was used to combine the results for all the genotypes. Then all the unfiltered genotypes were replaced in Paragraph software with missing genotypes (./.) and excluded SVs without any remaining nonreference genotypes.

Following the methodology outlined in a previous study,^[^ ^41^ ^]^ the midpoint of each SV was used to calculate SV hotspots via a publicly available script (https://github.com/daewoooo/primatR/blob/master/R/hotspotter.R) with minor modifications.

### Detection of Selective Signals

To detect SVs with highly differentiated allele frequencies between QTP cattle (Bailang, Dingjie, Diqing, Langkazi, and Yushu) and low‐altitude cattle (Fuzhou, Yanbian, Luxi and Simmental), the *d_i_* values for 222528 SVs were calculated from 84 LRS data as described in previous studies.^[^ ^43^, ^44^ ^]^ Chaidamu and Mongolian cattle were not included in either of the two groups because their altitudes were between 800 and 3000 m, which may have affected the results. For the SV‐*F*_ST_, the Weir and Cockerham estimator were employed for the *F*_ST_ estimates via VCFtools (v.0.1.16)^[^ ^112^ ^]^ to identify population‐stratified SVs in five groups: all QTP cattle versus low‐altitude cattle; Bailang versus low‐altitude cattle; Dingjie versus low‐altitude cattle; Diqing versus low‐altitude cattle; Langkazi versus low‐altitude cattle; and Yushu versus low‐altitude cattle. SVs with *P* values less than 0.001 (*Z* test) were selected as *d_i_*‐SVs.

### Annotation of SVs

Consistent sequences of SVs were extracted and compared with consistent repeat sequences of mammals using RepeatMasker (v.4.0.5) (http://www.repeatmasker.org) to determine whether they were repetitive sequences. Specifically, for DELs and DUPs, BEDTools (v.2.25.0)^[^ ^113^ ^]^ were used to intersect these segments with the repetitive sequences of the ARS‐UCD1.2, which requires a minimum reciprocal overlap of 80%. For INSs, the sequences were extracted and annotated using RepeatMasker. INSs were classified as repetitive sequences if the query sequence aligned completely, allowing for a maximum of 20 bp unaligned regions at the ends (left < 20). The functional regions of the SVs in the genomes were annotated using ANNOVAR.^[^ ^33^ ^]^ KEGG and GO enrichment analyses were performed for SV‐linked genes in population stratification, and functional categories with corrected *P* values less than 0.05 were considered significantly enriched.

### ATAC‐seq Analysis

Some of the ATAC‐seq data used in this study were generated through our own sequencing experiments, whereas others were obtained from publicly available NCBI datasets (Table S14, Supporting Information). After the adapters were checked for and trimmed with Trim Galore (v.0.6.10) (https://github.com/FelixKrueger/TrimGalore), the ATAC‐seq clean reads were mapped to ARS‐UCD1.2 using Bowtie2 (v.2.4.5).^[^ ^114^ ^]^ BAM files were sorted using SAMtools (v.1.3),^[^ ^115^ ^]^ and duplicate alignments were removed with Picard (v.2.20.2) (http://broadinstitute.github.io/picard). The peaks for individual replicates were called separately with MACS2 (v.2.2.7.1)^[^ ^116^ ^]^ and then merged across three duplicates within the same tissue using BEDTools (v.2.25.0).^[^ ^113^ ^]^ The R package obtained from https://github.com/junjunlab is used for drawing.

### Dual‐Luciferase Assay

For the 102‐bp DEL of the *GNPAT* gene, sequences containing the entire SV and the 360‐bp flanking sequences were extracted from the cattle genome (ARS‐UCD1.2) and inserted into the pGL3 promoter. The HEK293T cell line was purchased from the National Science & Technology Infrastructure (Shanghai, China). Transfection was performed when the cell density in the 12‐well plate reached approximately 80%. Lipofectamine 2000 (Invitrogen, CA, USA) was mixed with plasmids (recombinant plasmid and pRL‐TK plasmid vector) at a ratio of 1:1, incubated for 20 min, and then added to the cells. The dual‐luciferase reporter assay kit was purchased from Promega (Wisconsin, USA), and the experiments were conducted according to the manufacturer's instructions. After cell lysis, the lysates were transferred to a 96‐well plate, and two substrates were sequentially added. Detection was then performed using a Multiskan system (TECAN, Spark, Switzerland). Each experiment was independently performed three times. All the data are presented as the means with standard deviations, and significant differences between groups were examined via two‐sided Student's t test. A *P* value < 0.05 was considered to indicate a significant difference.

### Discovery of the Introgressed SVs

Yak LRS data were downloaded from the NCBI to identify SVs, including six domestic yak and 6 wild yak (Table S8, Supporting Information). Using SURVIVOR software, 12 yak and 84 cattle vcf files were merged, and the subsequent genotyping workflow was the same as that used for “SV discovery and genotyping”. After filtering out unreliable genotypes (all 0/0), a total of 250156 SVs was obtained. First, using LRS data, SVs specific to QTP cattle and fixed in yak genomes but absent from low‐altitude cattle populations (Fuzhou, Yanbian, Luxi, and Simmental) were investigated. A total of 7293 autosomal SVs possibly derived from yak introgression were identified in QTP cattle (Figure 4a). To further validate the SVs, haplotype trees were constructed using SNPs in the 50‐kb flanking of the SV, using a SNP dataset of 148 QTP (36 LRS and 112 SRS) samples and 16 reference SRS samples.

To expand the population size, the SVs were further genotyped using 293 SRS data, including 112 QTP cattle and 12 high‐coverage individuals from other bovine species (Table S16, Supporting Information). Paragraph (v.2.4a)^[^ ^111^ ^]^ was used to genotype the SVs from the SRS data. Cattle from low‐altitude taurine breeds/populations (African taurine cattle, European taurine cattle, and East Asian taurine cattle) were used as control populations for genotyping (Table S18, Supporting Information). Then SVs that were shared with yak genomes but not present in other cattle samples were searched, and 2334 candidate introgressed tract SVs remained after SRS genotyping. The –maf parameter of VCFtools (v.0.1.16)^[^ ^112^ ^]^ with the –freq parameter was used to calculate the allelic frequency in different breeds/populations. IQTREE (v.1.6.6)^[^ ^117^ ^]^ was used to construct a phylogenetic tree for the introgressed yak regions. ModelFinder^[^ ^118^ ^]^ was used to identify the best model of the phylogenetic tree.

### Hi‐C Data Preprocessing and Normalization

Hi‐C read pairs were processed using the HiC‐Pro pipeline^[^ ^119^ ^]^ with default parameters. After removing duplications, valid pairs were summed across three technical replicates and used to generate an interchromosome contact matrix. The normalized observed contact matrices were generated using the Knight‒Ruiz algorithm within the Juicer toolkit to eliminate intrinsic biases within the matrices. Quantile normalization was subsequently performed using the BNBC R package (v.1.0.0) to mitigate biases between samples. The correlation between normalized matrices of technical replicates was calculated using GenomeDISCO^[^ ^120^ ^]^ (Figure S20, Supporting Information).

### A/B Compartment Determination and Analysis

A/B compartments at 20 kb resolution were identified using PCA and the A‒B index as described in a previous study.^[^ ^121^ ^]^ In brief, observed/expected contact matrices were obtained by normalizing intrachromosomal observed contact matrices using KR normalization and quantile normalization. For loci *i* and *j*, the expected contact frequency was determined as the median observed contact frequency at the same genomic distance. The observed/expected contact matrices were then calculated by dividing the observed contact frequency by the expected contact frequency for each locus pair. The “prcomp” package was applied in R (v.4.1.2) with default parameters to these matrices to generate PC1 vectors. The gene density for each bin was defined as the number of transcriptional start sites (TSSs) within ±100 kb of the bin. Compartments A and B were identified by calculating the Pearson's correlation between PC1 values and gene density for each chromosome using the “cor.test” function in R (v.4.1.2). Positive PC1 values were assigned to compartment A, and negative values were assigned to compartment B if the correlation was positive. The assignment was reversed if the correlation was negative. The A‐B index was subsequently calculated at a 20 kb resolution following previously described methods, representing the likelihood of a sequence interacting with either the A or B compartment.^[^ ^121^ ^]^ Bins 20 kb in length with a positive A‐B index were classified as A compartments, whereas those with a negative A‐B index were classified as B compartments.

### TAD Identification and Loop Calling

TADs were identified via a previously reported insulation score (IS)^[^ ^122^ ^]^ method based on the KR and quantile‐normalized intrachromosomal observed contact matrices at a 20‐kb resolution. The IS reflects the aggregate interactions passing across each bin, and IS boundaries were identified using the public script matrix2insulation.pl (https://github.com/dekkerlab/cworld‐dekker) with the following parameters: ‐v, ‐is 260 000, ‐ids 200 000, ‐im mean, ‐nt 0.1, and ‐bmoe 0. Fit‐Hi‐C software (v.2.0.8)^[^ ^123^ ^]^ was used with default parameters to identify loops at a 5‐kb resolution based on the KR and quantile‐normalized intrachromosomal contact matrices, with a *q* value < 1×10^−8^ used as a cutoff.

### GWAS and F_ST_ Analysis of Gray Cattle

For both the SNP‐GWAS and SNP‐*F*_ST_ analyses, read mapping and SNP calling for the SRS data were performed as described in the previous “Variant calling evaluation” section. To identify the locus associated with gray coat color, SRS data from 23 gray cattle and 258 nongray cattle were used. SNP‐GWAS analysis was conducted on 62091927 SNPs using the GEMMA.^[^ ^124^ ^]^ The genome‐wide distribution of the *F*_ST_ values was estimated using VCFtools (v.0.1.16)^[^ ^112^ ^]^ with a 50‐kb window size and 25‐kb increment to examine pairwise genetic differentiation between 23 gray and 29 nongray cattle. For SV‐GWAS analysis, five gray cattle were used and the remaining 79 non‐gray cattle from the LRS dataset were analyzed with the GEMMA model. For SV‐*F*_ST_, five gray cattle and 11 non‐gray cattle were used to calculate the *F*_ST_ value for each SV.

### SV Validation

Visualization of the detected SVs was performed using IGV (v.2.2).^[^ ^125^ ^]^ To obtain more reliable detection outputs for inversions, npInv (v.1.24)^[^ ^68^ ^]^ was used to detect and genotype inversion variants. To further verify the authenticity and heterozygosity of the 2‐Mb INV and two associated TRAs, breakpoint‐spanning primers were designed and PCR validation was performed in an additional 23 gray cattle. Three primer pairs were used to independently validate each SV: Primer1 (F: 5′‐GTTGACCCTTTCTTTCCA‐3′; R: 5′‐GACCCTCCTTTCTATCCC‐3′) targeted TRA (BTA17:47058249‐BTA6:69764591); Primer2 (F: 5′‐ATCTTAGCTGTAGCATGT‐3′; R: 5′‐ACTTGTAGGTTACTGGTT‐3′) spanned the INV (BTA6:69722181‐71822202) breakpoints; and Primer3 (F: 5′‐TCCTTCCCTAAGATTCAGA‐3′; R: 5′‐GAGCCCTTGACCCACTA‐3′) amplified TRA (BTA6:70471991‐BTA17:46961621).

For the four introgressed SVs, four primer pairs were designed: Primer4 (F: 5′‐AAGACGCTTGCTCC‐3′; R: 5′‐CTTCTCCAAAACCACA‐3′) targeted DEL (BTA28:4236113‐4237437); Primer5 (F: 5′‐CTGGTCCCATCACTTC‐3′; R: 5′‐TTGCCATACCTCATTTT‐3′) spanned the INS (BTA28:4204966) breakpoint; Primer6 (F: 5′‐CTTAGGACTTCAGGGA‐3′; R: 5′‐GCTCATCATAGGTTTGTA‐3′) amplified DEL (BTA9:87336847‐87336904); and Primer7 (F: 5′‐ACCCTGTACCTCTTAGCA‐3′; R: 5′‐GACATGACTGAGCGACTG‐3′) targeted DEL (BTA2:19540699‐19540751) (Figure S21, Supporting Information).

PCR was performed in 25 µL reactions with 1 µL of genomic DNA (50 ng), 1 µL each of forward and reverse forward primers (10 µM), and 22 µL of Golden Star T6 Super PCR mix (Beijing Tsingke Biotech Co., Beijing, China). The PCR products were examined using 1.5% agarose gel electrophoresis. The presence and size of the amplified fragments were determined and used to infer the genotype of the inversion.

### Immunohistochemistry and H&E Staining

Skin samples were fixed in 4% paraformaldehyde, washed in PBS, dehydrated and embedded in paraffin. The rabbit polyclonal antibody anti‐KIT (D260893, Sangon) was used for immunohistochemical staining.

### Linkage Disequilibrium

LD was accessed across the chromosome containing the 2‐Mb INV (BTA6:69770000‐71780000) and the extended region (BTA6:67770000‐73780000) using samples from both gray and non‐gray cattle. Gray cattle group: comprising 28 samples from Dingjie (n = 13), Langkazi (n = 14), and Bailang cattle (n = 1) in the QTP. Non‐gray cattle group: consisting of Dingjie (n = 29), Diqing (n = 19), and Yushu cattle (n = 32) from the QTP. Randomly 8000 SNPs from the VCF file were sampled and haplotype blocks were analyzed using LDBlockShow^[^ ^126^ ^]^ with the parameter ‘‐SeleVar 2′.

## Conflict of Interest

The authors declare no conflict of interest.

## Author Contributions

X.X., F.W., X.L., S.L. and L.Y. contributed equally to this work. N.C., C.L., W.L., and B.H. designed and supervised the project. X.X., F.W., X.L., S.L. and L.Y. performed most of the analyses. K.Q., R.S., J.L., J.Z., B.W., B.Z., S.Q., L.Z., S.W., C. Luobu, N. Cangjue, D.L., S.S., Z.M., R.L., Z.W., X.Y., and H.C. collected the samples. Y.Z. and L.D. conducted the experiments. X.X., F.W. and N.C. wrote the manuscript with input from all the authors. N.C., J.A.L., J.H., L.X., H.H., W.L., Z.Z., Y.W., Y.G., R.D., Y.H. and X.L. revised the manuscript.

## Supporting information



Supporting Information

Supplemental Table 1

## Data Availability

The data that support the findings of this study are openly available in NCBI at https://dataview.ncbi.nlm.nih.gov/object/PRJNA1158668?reviewer=k781773a0ej2l1mnj2j9j41a8l, reference number 1158668.

## References

[1] Q. Qiu , G. Zhang , T. Ma , W. Qian , J. Wang , Z. Ye , C. Cao , Q. Hu , J. Kim , D. M. Larkin , L. Auvil , B. Capitanu , J. Ma , H. A. Lewin , X. Qian , Y. Lang , R. Zhou , L. Wang , K. Wang , J. Xia , S. Liao , S. Pan , X. Lu , H. Hou , Y. Wang , X. Zang , Y. Yin , H. Ma , J. Zhang , Z. Wang , et al., Nat. Genet. 2012, 44, 946. 22751099 10.1038/ng.2343

[2] G. D. Wang , R. X. Fan , W. Zhai , F. Liu , L. Wang , L. Zhong , H. Wu , H. C. Yang , S. F. Wu , C. L. Zhu , Y. Li , Y. Gao , R. L. Ge , C. I. Wu , Y. P. Zhang , Genome Biol Evol 2014, 6, 2122. 25091388 10.1093/gbe/evu162 PMC4231634

[3] X. Gou , Z. Wang , N. Li , F. Qiu , Z. Xu , D. Yan , S. Yang , J. Jia , X. Kong , Z. Wei , S. Lu , L. Lian , C. Wu , X. Wang , G. Li , T. Ma , Q. Jiang , X. Zhao , J. Yang , B. Liu , D. Wei , H. Li , J. Yang , Y. Yan , G. Zhao , X. Dong , M. Li , W. Deng , J. Leng , C. Wei , et al., Genome Res. 2014, 24, 1308. 24721644 10.1101/gr.171876.113 PMC4120084

[4] X. Liu , Y. Zhang , Y. Li , J. Pan , D. Wang , W. Chen , Z. Zheng , X. He , Q. Zhao , Y. Pu , W. Guan , J. Han , L. Orlando , Y. Ma , L. Jiang , Mol. Biol. Evol. 2019, 36, 2591. 31273382 10.1093/molbev/msz158 PMC6805228

[5] M. Li , S. Tian , L. Jin , G. Zhou , Y. Li , Y. Zhang , T. Wang , C. K. Yeung , L. Chen , J. Ma , J. Zhang , A. Jiang , J. Li , C. Zhou , J. Zhang , Y. Liu , X. Sun , H. Zhao , Z. Niu , P. Lou , L. Xian , X. Shen , S. Liu , S. Zhang , M. Zhang , L. Zhu , S. Shuai , L. Bai , G. Tang , H. Liu , et al., Nat. Genet. 2013, 45, 1431. 24162736 10.1038/ng.2811

[6] M. S. Wang , Y. Li , M. S. Peng , L. Zhong , Z. J. Wang , Q. Y. Li , X. L. Tu , Y. Dong , C. L. Zhu , L. Wang , M. M. Yang , S. F. Wu , Y. W. Miao , J. P. Liu , D. M. Irwin , W. Wang , D. D. Wu , Y. P. Zhang , Mol. Biol. Evol. 2015, 32, 1880. 25788450 10.1093/molbev/msv071

[7] D. D. Wu , C. P. Yang , M. S. Wang , K. Z. Dong , D. W. Yan , Z. Q. Hao , S. Q. Fan , S. Z. Chu , Q. S. Shen , L. P. Jiang , Y. Li , L. Zeng , H. Q. Liu , H. B. Xie , Y. F. Ma , X. Y. Kong , S. L. Yang , X. X. Dong , A. Esmailizadeh , D. M. Irwin , X. Xiao , M. Li , Y. Dong , W. Wang , P. Shi , H. P. Li , Y. H. Ma , X. Gou , Y. B. Chen , Y. P. Zhang , Natl. Sci. Rev. 2020, 7, 952. 34692117 10.1093/nsr/nwz213 PMC8288980

[8] W. Zhang , Z. Fan , E. Han , R. Hou , L. Zhang , M. Galaverni , J. Huang , H. Liu , P. Silva , P. Li , J. P. Pollinger , L. Du , X. Zhang , B. Yue , R. K. Wayne , Z. Zhang , PLoS Genet. 2014, 10, 1004466. 10.1371/journal.pgen.1004466 PMC4117439 25078401

[9] T. S. Simonson , Y. Yang , C. D. Huff , H. Yun , G. Qin , D. J. Witherspoon , Z. Bai , F. R. Lorenzo , J. Xing , L. B. Jorde , J. T. Prchal , R. Ge , Science 2010, 329, 72. 20466884 10.1126/science.1189406

[10] R. T. Loftus , D. E. MacHugh , D. G. Bradley , P. M. Sharp , P. Cunningham , Proc Natl Acad Sci U S A 91, 2757. 10.1073/pnas.91.7.2757 PMC43449 8146187

[11] J.‐D. Vigne , J. Peters , D. Helmer , The first steps of animal domestication, Oxbow Books, Oxford 2005.

[12] J. E. Decker , S. D. McKay , M. M. Rolf , J. Kim , A. Molina Alcalá , T. S. Sonstegard , O. Hanotte , A. Götherström , C. M. Seabury , L. Praharani , M. E. Babar , L. Correia de Almeida Regitano , M. A. Yildiz , M. P. Heaton , W. S. Liu , C. Z. Lei , J. M. Reecy , M. Saif‐Ur‐Rehman , R. D. Schnabel , J. F. Taylor , PLoS Genet. 2014, 10, 1004254. 10.1371/journal.pgen.1004254 PMC3967955 24675901

[13] N. Chen , Y. Cai , Q. Chen , R. Li , K. Wang , Y. Huang , S. Hu , S. Huang , H. Zhang , Z. Zheng , W. Song , Z. Ma , Y. Ma , R. Dang , Z. Zhang , L. Xu , Y. Jia , S. Liu , X. Yue , W. Deng , X. Zhang , Z. Sun , X. Lan , J. Han , H. Chen , D. G. Bradley , Y. Jiang , C. Lei , Nat. Commun. 2018, 9, 2337. 29904051 10.1038/s41467-018-04737-0 PMC6002414

[14] D. D. Wu , X. D. Ding , S. Wang , J. M. Wójcik , Y. Zhang , M. Tokarska , Y. Li , M. S. Wang , O. Faruque , R. Nielsen , Q. Zhang , Y. P. Zhang , Nat Ecol Evol 2018, 2, 1139. 29784979 10.1038/s41559-018-0562-y

[15] I. Medugorac , A. Graf , C. Grohs , S. Rothammer , Y. Zagdsuren , E. Gladyr , N. Zinovieva , J. Barbieri , D. Seichter , I. Russ , A. Eggen , G. Hellenthal , G. Brem , H. Blum , S. Krebs , A. Capitan , Nat. Genet. 2017, 49, 470. 28135247 10.1038/ng.3775

[16] K. Zhang , J. A. Lenstra , S. Zhang , W. Liu , J. Liu , Anim Genet 2020, 51, 637. 32716565 10.1111/age.12974

[17] X. T. Xia , A. Achilli , J. A. Lenstra , B. Tong , Y. Ma , Y. Z. Huang , J. L. Han , Z. Y. Sun , H. Chen , C. Z. Lei , S. M. Hu , N. B. Chen , Heredity (Edinb) 2021, 126, 1000. 33782560 10.1038/s41437-021-00428-7 PMC8178343

[18] N. Chen , X. Xia , Q. Hanif , F. Zhang , R. Dang , B. Huang , Y. Lyu , X. Luo , H. Zhang , H. Yan , S. Wang , F. Wang , J. Chen , X. Guan , Y. Liu , S. Li , L. Jin , P. Wang , L. Sun , J. Zhang , J. Liu , K. Qu , Y. Cao , J. Sun , Y. Liao , Z. Xiao , M. Cai , L. Mu , A. Z. Siddiki , M. Asif , et al., Nat. Commun. 2023, 14, 7803. 38016956 10.1038/s41467-023-43626-z PMC10684552

[19] Y. Lyu , F. Wang , H. Cheng , J. Han , R. Dang , X. Xia , H. Wang , J. Zhong , J. A. Lenstra , H. Zhang , J. Han , D. E. MacHugh , I. Medugorac , M. Upadhyay , A. S. Leonard , H. Ding , X. Yang , M. S. Wang , S. Quji , B. Zhuzha , P. Quzhen , S. Wangmu , N. Cangjue , D. Wa , W. Ma , J. Liu , J. Zhang , B. Huang , X. Qi , F. Li , et al., Sci. Bull. (Beijing) 2024, 69, 3415. 38945748 10.1016/j.scib.2024.05.030

[20] Y. He , H. Lou , C. Cui , L. Deng , Y. Gao , W. Zheng , Y. Guo , X. Wang , Z. Ning , J. Li , B. Li , C. Bai , S. Liu , T. Wu , S. Xu , X. Qi , B. Su , Natl. Sci. Rev. 2020, 7, 391. 34692055 10.1093/nsr/nwz160 PMC8288928

[21] C. Chiang , A. J. Scott , J. R. Davis , E. K. Tsang , X. Li , Y. Kim , T. Hadzic , F. N. Damani , L. Ganel , S. B. Montgomery , A. Battle , D. F. Conrad , I. M. Hall , Nat. Genet. 2017, 49, 692. 28369037 10.1038/ng.3834 PMC5406250

[22] C. Quan , Y. Li , X. Liu , Y. Wang , J. Ping , Y. Lu , G. Zhou , Genome Biol. 2021, 22, 159. 34034800 10.1186/s13059-021-02382-3 PMC8146648

[23] J. A. Lenstra , Animal Research and One Health 2024, 2, 360.

[24] X. Li , Q. Liu , C. Fu , M. Li , C. Li , X. Li , S. Zhao , Z. Zheng , J Genet Genomics 2024, 51, 394. 38056526 10.1016/j.jgg.2023.11.005

[25] H. Cheng , G. T. Concepcion , X. Feng , H. Zhang , H. Li , Nat. Methods 2021, 18, 170. 33526886 10.1038/s41592-020-01056-5 PMC7961889

[26] B. D. Ondov , T. J. Treangen , P. Melsted , A. B. Mallonee , N. H. Bergman , S. Koren , A. M. Phillippy , Genome Biol. 2016, 17, 132. 27323842 10.1186/s13059-016-0997-x PMC4915045

[27] A. Rhie , B. P. Walenz , S. Koren , A. M. Phillippy , Genome Biol. 2020, 21, 245. 32928274 10.1186/s13059-020-02134-9 PMC7488777

[28] H. Lou , Y. Gao , B. Xie , Y. Wang , H. Zhang , M. Shi , S. Ma , X. Zhang , C. Liu , S. Xu , Cell Syst 2022, 13, 321. 35180379 10.1016/j.cels.2022.01.006

[29] S. Schiffels , R. Durbin , Nat. Genet. 2014, 46, 919. 24952747 10.1038/ng.3015 PMC4116295

[30] T. Jiang , Y. Liu , Y. Jiang , J. Li , Y. Gao , Z. Cui , Y. Liu , B. Liu , Y. Wang , Genome Biol. 2020, 21, 189. 32746918 10.1186/s13059-020-02107-y PMC7477834

[31] D. Heller , M. Vingron , Bioinformatics 2019, 35, 2907. 30668829 10.1093/bioinformatics/btz041 PMC6735718

[32] F. J. Sedlazeck , P. Rescheneder , M. Smolka , H. Fang , M. Nattestad , A. von Haeseler , M. C. Schatz , Nat. Methods 2018, 15, 461. 29713083 10.1038/s41592-018-0001-7 PMC5990442

[33] K. Wang , M. Li , H. Hakonarson , Nucleic Acids Res. 2010, 38, 164. 10.1093/nar/gkq603 PMC2938201 20601685

[34] X. Dai , P. Bian , D. Hu , F. Luo , Y. Huang , S. Jiao , X. Wang , M. Gong , R. Li , Y. Cai , J. Wen , Q. Yang , W. Deng , H. A. Nanaei , Y. Wang , F. Wang , Z. Zhang , B. D. Rosen , R. Heller , Y. Jiang , Genome Res. 2023, 33, 1284. 37714713 10.1101/gr.277481.122 PMC10547261

[35] C. J. Kelly , C. G. Chitko‐McKown , E. B. Chuong , Genome Res. 2022, 32, 1474. 35948370 10.1101/gr.276241.121 PMC9435751

[36] A. S. Leonard , X. M. Mapel , H. Pausch , Genome Res. 2024, 34, 300. 38355307 10.1101/gr.278267.123 PMC10984387

[37] R. Li , M. Gong , X. Zhang , F. Wang , Z. Liu , L. Zhang , Q. Yang , Y. Xu , M. Xu , H. Zhang , Y. Zhang , X. Dai , Y. Gao , Z. Zhang , W. Fang , Y. Yang , W. Fu , C. Cao , P. Yang , Z. A. Ghanatsaman , N. J. Negari , H. A. Nanaei , X. Yue , Y. Song , X. Lan , W. Deng , X. Wang , C. Pan , R. Xiang , E. M. Ibeagha‐Awemu , et al., Genome Res. 2023, 33, 463. 37310928 10.1101/gr.277372.12 PMC10078295

[38] Z. Li , X. Liu , C. Wang , Z. Li , B. Jiang , R. Zhang , L. Tong , Y. Qu , S. He , H. Chen , Y. Mao , Q. Li , T. Pook , Y. Wu , Y. Zan , H. Zhang , L. Li , K. Wen , Y. Chen , Genome Res. 2023, 33, 1833. 37914227 10.1101/gr.277638.122 PMC10691484

[39] D. Crysnanto , A. S. Leonard , Z. H. Fang , H. Pausch , Proc Natl Acad Sci U S A 2021, 118, 2101056118. 10.1073/pnas.2101056118 PMC8157972 33972446

[40] A. Talenti , J. Powell , J. D. Hemmink , E. A. J. Cook , D. Wragg , S. Jayaraman , E. Paxton , C. Ezeasor , E. T. Obishakin , E. R. Agusi , A. Tijjani , K. Marshall , A. Fisch , B. R. Ferreira , A. Qasim , U. Chaudhry , P. Wiener , P. Toye , L. J. Morrison , T. Connelley , J. G. D. Prendergast , Nat. Commun. 2022, 13, 910. 35177600 10.1038/s41467-022-28605-0 PMC8854726

[41] P. Ebert , P. A. Audano , Q. Zhu , B. Rodriguez‐Martin , D. Porubsky , M. J. Bonder , A. Sulovari , J. Ebler , W. Zhou , R. Serra Mari , F. Yilmaz , X. Zhao , P. Hsieh , J. Lee , S. Kumar , J. Lin , T. Rausch , Y. Chen , J. Ren , M. Santamarina , W. Höps , H. Ashraf , N. T. Chuang , X. Yang , K. M. Munson , A. P. Lewis , S. Fairley , L. J. Tallon , W. E. Clarke , A. O. Basile , et al., Science 2021, 372, abf7117. 10.1126/science.abf7117 PMC8026704 33632895

[42] T. T. Li , T. Xia , J. Q. Wu , H. Hong , Z. L. Sun , M. Wang , F. R. Ding , J. Wang , S. Jiang , J. Li , J. Pan , G. Yang , J. N. Feng , Y. P. Dai , X. M. Zhang , T. Zhou , T. Li , Nat. Commun. 2023, 14, 6601. 37857610 10.1038/s41467-023-42161-1 PMC10587341

[43] J. M. Akey , A. L. Ruhe , D. T. Akey , A. K. Wong , C. F. Connelly , J. Madeoy , T. J. Nicholas , M. W. Neff , Proc Natl Acad Sci U S A 2010, 107, 1160. 20080661 10.1073/pnas.0909918107 PMC2824266

[44] Y. Feng , N. Xie , F. Inoue , S. Fan , J. Saskin , C. Zhang , F. Zhang , M. E. B. Hansen , T. Nyambo , S. W. Mpoloka , G. G. Mokone , C. Fokunang , G. Belay , A. K. Njamnshi , M. S. Marks , E. Oancea , N. Ahituv , S. A. Tishkoff , Nat. Genet. 2024, 56, 258. 38200130 10.1038/s41588-023-01626-1 PMC11005318

[45] W. Zheng , Y. He , Y. Guo , T. Yue , H. Zhang , J. Li , B. Zhou , X. Zeng , L. Li , B. Wang , J. Cao , L. Chen , C. Li , H. Li , C. Cui , C. Bai , Baimakangzhuo , X. Qi , Ouzhuluobu , B. Su , Genome Biol. 2023, 24, 73. 37055782 10.1186/s13059-023-02912-1 PMC10099689

[46] R. A. Torres , D. A. Drake , V. Solodushko , R. Jadhav , E. Smith , P. Rocic , D. S. Weber , Arterioscler Thromb Vasc Biol 2011, 31, 2424. 21868701 10.1161/ATVBAHA.111.232769 PMC3204790

[47] Y. Wang , X. Liu , B. Xie , H. Yuan , Y. Zhang , J. Zhu , Redox Biol. 2020, 28, 101313. 31539803 10.1016/j.redox.2019.101313 PMC6812007

[48] L. Cocco , L. Manzoli , I. Faenza , G. Ramazzotti , Y. R. Yang , J. A. McCubrey , P. G. Suh , M. Y. Follo , Adv. Biol. Regul. 2016, 60, 1. 26525203 10.1016/j.jbior.2015.10.008

[49] M. A. S. Pinhal , C. M. Melo , H. B. Nader , Adv. Exp. Med. Biol. 2020, 1221, 821. 32274740 10.1007/978-3-030-34521-1_36

[50] M. Elkin , N. Ilan , R. Ishai‐Michaeli , Y. Friedmann , O. Papo , I. Pecker , I. Vlodavsky , FASEB J. 2001, 15, 1661. 11427519 10.1096/fj.00-0895fje

[51] P. Zhao , F. Zhao , J. Hu , J. Wang , X. Liu , Z. Zhao , Q. Xi , H. Sun , S. Li , Y. Luo , Front Physiol 2022, 13, 885444. 35634140 10.3389/fphys.2022.885444 PMC9133604

[52] S. Zhu , T. Guo , H. Zhao , G. Qiao , M. Han , J. Liu , C. Yuan , T. Wang , F. Li , Y. Yue , B. Yang , Front. Genet. 2020, 11, 848. 32849829 10.3389/fgene.2020.00848 PMC7411260

[53] J. Hu , M. R. Hameed , N. P. Agaram , K. A. Whiting , L. X. Qin , A. M. Villano , R. B. O'Connor , J. M. Rozenberg , S. Cohen , K. Prendergast , S. Kryeziu , R. L. White, Jr. , M. C. Posner , N. D. Socci , M. M. Gounder , S. Singer , A. M. Crago , Clin. Cancer Res. 2024, 30, 450. 37943631 10.1158/1078-0432.CCR-23-2313 PMC10792363

[54] I. H. Jain , S. E. Calvo , A. L. Markhard , O. S. Skinner , T. L. To , T. Ast , V. K. Mootha , Cell 2020, 181, 716. 32259488 10.1016/j.cell.2020.03.029 PMC7293541

[55] W. H. Tang , S. Wu , T. M. Wong , S. K. Chung , S. S. Chung , Free Radic Biol Med 2008, 45, 602. 18549825 10.1016/j.freeradbiomed.2008.05.003

[56] T. L. Clanton , J. Appl. Physiol. 2007, 102, 2379. 17289907 10.1152/japplphysiol.01298.2006

[57] Z. Wu , M. Zuo , L. Zeng , K. Cui , B. Liu , C. Yan , L. Chen , J. Dong , F. Shangguan , W. Hu , H. He , B. Lu , Z. Song , EMBO Rep. 2021, 22, 50827. 10.15252/embr.202050827 PMC7788456 33314701

[58] T. Wang , E. Wiater , X. Zhang , J. B. Thomas , M. Montminy , Proc Natl Acad Sci U S A 2021, 118. 10.1073/pnas.2024865118 PMC8000278 33723074

[59] N. Maeda , T. Funahashi , Y. Matsuzawa , I. Shimomura , Atherosclerosis 2020, 292, 1. 31731079 10.1016/j.atherosclerosis.2019.10.021

[60] Z. Chen , I. L. Ho , M. Soeung , E. Y. Yen , J. Liu , L. Yan , J. L. Rose , S. Srinivasan , S. Jiang , Q. E. Chang , N. Feng , J. P. Gay , Q. Wang , J. Wang , P. L. Lorenzi , L. J. Veillon , B. Wei , J. N. Weinstein , A. K. Deem , S. Gao , G. Genovese , A. Viale , W. Yao , C. A. Lyssiotis , J. R. Marszalek , G. F. Draetta , H. Ying , Nat. Commun. 2023, 14, 2194. 37069167 10.1038/s41467-023-37924-9 PMC10110566

[61] J. Han , D. Zheng , P. S. Liu , S. Wang , X. Xie , Cell Commun Signal 2024, 22, 475. 39367496 10.1186/s12964-024-01862-w PMC11451054

[62] H. S. Radomska , C. S. Huettner , P. Zhang , T. Cheng , D. T. Scadden , D. G. Tenen , Mol. Cell. Biol. 1998, 18, 4301. 9632814 10.1128/mcb.18.7.4301 PMC109014

[63] X. Liu , W. Liu , J. A. Lenstra , Z. Zheng , X. Wu , J. Yang , B. Li , Y. Yang , Q. Qiu , H. Liu , K. Li , C. Liang , X. Guo , X. Ma , R. J. Abbott , M. Kang , P. Yan , J. Liu , Nat. Commun. 2023, 14, 5617. 37726270 10.1038/s41467-023-41220-x PMC10509194

[64] C. Li , B. Chen , S. Langda , P. Pu , X. Zhu , S. Zhou , P. Kalds , K. Zhang , M. Bhati , A. Leonard , S. Huang , R. Li , A. Cuoji , X. Wang , H. Zhu , Y. Wu , R. Cuomu , B. Gui , M. Li , Y. Wang , Y. Li , W. Fang , T. Jia , T. Pu , X. Pan , Y. Cai , C. He , L. Wang , Y. Jiang , J. L. Han , et al., Genomics Proteomics Bioinformatics 2024, 22. 10.1093/gpbjnl/qzae030 PMC12016566 39142817

[65] K. Tsuji‐Tamura , M. Ogawa , Angiogenesis 2023, 26, 523. 37488325 10.1007/s10456-023-09884-7

[66] J. Zheng , S. J. Kim , S. Saeidi , S. H. Kim , X. Fang , Y. H. Lee , Y. N. Guillen‐Quispe , H. K. C. Ngo , D. H. Kim , D. Kim , Y. J. Surh , Free Radic Biol Med 2023, 194, 347. 36460215 10.1016/j.freeradbiomed.2022.11.039

[67] G. R. Pielberg , A. Golovko , E. Sundström , I. Curik , J. Lennartsson , M. H. Seltenhammer , T. Druml , M. Binns , C. Fitzsimmons , G. Lindgren , K. Sandberg , R. Baumung , M. Vetterlein , S. Strömberg , M. Grabherr , C. Wade , K. Lindblad‐Toh , F. Pontén , C. H. Heldin , J. Sölkner , L. Andersson , Nat. Genet. 2008, 40, 1004. 18641652 10.1038/ng.185

[68] H. Shao , D. Ganesamoorthy , T. Duarte , M. D. Cao , C. J. Hoggart , L. J. M. Coin , BMC Bioinformatics 2018, 19, 261. 30001702 10.1186/s12859-018-2252-9 PMC6044046

[69] E. Kabirova , A. Ryzhkova , V. Lukyanchikova , A. Khabarova , A. Korablev , T. Shnaider , M. Nuriddinov , P. Belokopytova , A. Smirnov , N. V. Khotskin , G. Kontsevaya , I. Serova , N. Battulin , Nat. Commun. 2024, 15, 4521. 38806452 10.1038/s41467-024-48523-7 PMC11133455

[70] W. Y. Low , Animal Research and One Health 2024, 2, 363.

[71] W. F. Blazak , F. E. Eldridge , J. Dairy Sci. 1977, 60, 1133. 881476 10.3168/jds.S0022-0302(77)83999-4

[72] A. S. Leonard , D. Crysnanto , Z. H. Fang , M. P. Heaton , B. L. Vander Ley , C. Herrera , H. Bollwein , D. M. Bickhart , K. L. Kuhn , T. P. L. Smith , B. D. Rosen , H. Pausch , Nat. Commun. 2022, 13, 3012. 35641504 10.1038/s41467-022-30680-2 PMC9156671

[73] A. Mandefro , T. Sisay , Z. Edea , M. R. Uzzaman , K. S. Kim , H. Dadi , J Anim Sci Technol 2021, 63, 248. 33987601 10.5187/jast.2021.e37 PMC8071750

[74] S. N. Takeshima , C. Corbi‐Botto , G. Giovambattista , Y. Aida , BMC Genet. 2018, 19, 33. 29788904 10.1186/s12863-018-0618-7 PMC5964877

[75] C. Li , Y. Wu , B. Chen , Y. Cai , J. Guo , A. S. Leonard , P. Kalds , S. Zhou , J. Zhang , P. Zhou , S. Gan , T. Jia , T. Pu , L. Suo , Y. Li , K. Zhang , L. Li , M. Purevdorj , X. Wang , M. Li , Y. Wang , Y. Liu , S. Huang , T. Sonstegard , M. S. Wang , S. Kemp , H. Pausch , Y. Chen , J. L. Han , Y. Jiang , et al., Mol. Biol. Evol. 2022, 39. 10.1093/molbev/msac253 PMC9728798 36382357

[76] X. Xia , F. Zhang , S. Li , X. Luo , L. Peng , Z. Dong , H. Pausch , A. S. Leonard , D. Crysnanto , S. Wang , B. Tong , J. A. Lenstra , J. Han , F. Li , T. Xu , L. Gu , L. Jin , R. Dang , Y. Huang , X. Lan , G. Ren , Y. Wang , Y. Gao , Z. Ma , H. Cheng , Y. Ma , H. Chen , W. Pang , C. Lei , N. Chen , Genome Biol. 2023, 24, 211. 37723525 10.1186/s13059-023-03052-2 PMC10507960

[77] L. Deng , C. Zhang , K. Yuan , Y. Gao , Y. Pan , X. Ge , Y. He , Y. Yuan , Y. Lu , X. Zhang , H. Chen , H. Lou , X. Wang , D. Lu , J. Liu , L. Tian , Q. Feng , A. Khan , Y. Yang , Z. B. Jin , J. Yang , F. Lu , J. Qu , L. Kang , B. Su , S. Xu , Natl. Sci. Rev. 2019, 6, 1201. 34691999 10.1093/nsr/nwz108 PMC8291452

[78] Q. Qiu , L. Wang , K. Wang , Y. Yang , T. Ma , Z. Wang , X. Zhang , Z. Ni , F. Hou , R. Long , R. Abbott , J. Lenstra , J. Liu , Nat. Commun. 2015, 6, 10283. 26691338 10.1038/ncomms10283 PMC4703879

[79] G. L. Semenza , E. A. Rue , N. V. Iyer , M. G. Pang , W. G. Kearns , Genomics 1996, 34, 437. 8786149 10.1006/geno.1996.0311

[80] C. W. Pugh , P. J. Ratcliffe , Nat. Med. 2003, 9, 677. 12778166 10.1038/nm0603-677

[81] J. Si , J. Guo , X. Zhang , W. Li , S. Zhang , S. Shang , Q. Zhang , Biol. Direct 2024, 19, 45. 38863009 10.1186/s13062-024-00487-w PMC11165725

[82] B. Borsari , P. Villegas‐Mirón , S. Pérez‐Lluch , I. Turpin , H. Laayouni , A. Segarra‐Casas , J. Bertranpetit , R. Guigó , S. Acosta , Genome Res. 2021, 31, 1325. 34290042 10.1101/gr.270371.120 PMC8327915

[83] Z. Yang , C. Bai , Y. Pu , Q. Kong , Y. Guo , X. Liu , Q. Zhao , Z. Qiu , W. Zheng , Y. He , Y. Lin , L. Deng , C. Zhang , S. Xu , Y. Peng , K. Xiang , X. Zhang , C. Cui , Y. Pan , J. Xin , Y. Wang , S. Liu , L. Wang , H. Guo , Z. Feng , S. Wang , H. Shi , B. Jiang , T. Wu , X. Qi , et al., Proc Natl Acad Sci U S A 2022, 119, 2200421119. 10.1073/pnas.2200421119 PMC9552612 36161951

[84] J. F. Storz , Mol. Biol. Evol. 2021, 38, 2677. 33751123 10.1093/molbev/msab064 PMC8233491

[85] X. An , L. Mao , Y. Wang , Q. Xu , X. Liu , S. Zhang , Z. Qiao , B. Li , F. Li , Z. Kuang , N. Wan , X. Liang , Q. Duan , Z. Feng , X. Yang , S. Liu , E. Nevo , J. Liu , J. F. Storz , K. Li , Nat Ecol Evol 2024, 8, 339. 38195998 10.1038/s41559-023-02275-7 PMC12301030

[86] J. Shi , Z. Jia , J. Sun , X. Wang , X. Zhao , C. Zhao , F. Liang , X. Song , J. Guan , X. Jia , J. Yang , Q. Chen , K. Yu , Q. Jia , J. Wu , D. Wang , Y. Xiao , X. Xu , Y. Liu , S. Wu , Q. Zhong , J. Wu , S. Cui , X. Bo , Z. Wu , M. Park , M. Kellis , K. He , Nat. Commun. 2023, 14, 8282. 38092772 10.1038/s41467-023-44034-z PMC10719358

[87] T. Bae , S. P. Hallis , M. K. Kwak , Exp. Mol. Med. 2024, 56, 501. 38424190 10.1038/s12276-024-01180-8 PMC10985007

[88] X. Ge , Y. Lu , S. Chen , Y. Gao , L. Ma , L. Liu , J. Liu , X. Ma , L. Kang , S. Xu , Mol. Biol. Evol. 2023, 40, msad205. 37713634 10.1093/molbev/msad205 PMC10584363

[89] I. Said , A. Byrne , V. Serrano , C. Cardeno , C. Vollmers , R. Corbett‐Detig , Proc Natl Acad Sci U S A 2018, 115, 5492. 29735663 10.1073/pnas.1721275115 PMC6003460

[90] K. Bilgrav Saether , J. Eisfeldt , J. D. Bengtsson , M. Y. Lun , C. M. Grochowski , M. Mahmoud , H. T. Chao , J. A. Rosenfeld , P. Liu , M. Ek , J. Schuy , A. Ameur , H. Dai , J. P. Hwang , F. J. Sedlazeck , W. Bi , R. Marom , J. Wincent , A. Nordgren , C. M. B. Carvalho , A. Lindstrand , Genome Res. 2024, 34, 1785. 39486878 10.1101/gr.279346.124 PMC11610578

[91] D. Nilsson , M. Pettersson , P. Gustavsson , A. Förster , W. Hofmeister , J. Wincent , V. Zachariadis , B. M. Anderlid , A. Nordgren , O. Mäkitie , V. Wirta , M. Käller , F. Vezzi , J. R. Lupski , M. Nordenskjöld , E. S. Lundberg , C. M. B. Carvalho , A. Lindstrand , Hum Mutat 2017, 38, 180. 27862604 10.1002/humu.23146 PMC5225243

[92] J. Guan , Y. Xu , Y. Yu , J. Fu , F. Ren , J. Guo , J. Zhao , Q. Jiang , J. Wei , H. Xie , Genome Biol. 2021, 22, 13. 33402202 10.1186/s13059-020-02239-1 PMC7784018

[93] O. S. Harringmeyer , H. E. Hoekstra , Nat Ecol Evol 2022, 6, 1965. 36253543 10.1038/s41559-022-01890-0 PMC9715431

[94] P. J. Hansen , Anim. Reprod. Sci. 2004, 82–83, 349. 10.1016/j.anireprosci.2004.04.011 15271465

[95] M. D. Littlejohn , K. M. Henty , K. Tiplady , T. Johnson , C. Harland , T. Lopdell , R. G. Sherlock , W. Li , S. D. Lukefahr , B. C. Shanks , D. J. Garrick , R. G. Snell , R. J. Spelman , S. R. Davis , Nat. Commun. 2014, 5, 5861. 25519203 10.1038/ncomms6861 PMC4284646

[96] J. Plassais , J. Kim , B. W. Davis , D. M. Karyadi , A. N. Hogan , A. C. Harris , B. Decker , H. G. Parker , E. A. Ostrander , Nat. Commun. 2019, 10, 1489. 30940804 10.1038/s41467-019-09373-w PMC6445083

[97] S. S. Rao , M. H. Huntley , N. C. Durand , E. K. Stamenova , I. D. Bochkov , J. T. Robinson , A. L. Sanborn , I. Machol , A. D. Omer , E. S. Lander , E. L. Aiden , Cell 2014, 159, 1665. 25497547 10.1016/j.cell.2014.11.021 PMC5635824

[98] N. C. Durand , M. S. Shamim , I. Machol , S. S. Rao , M. H. Huntley , E. S. Lander , E. L. Aiden , Cell Syst 2016, 3, 95. 27467249 10.1016/j.cels.2016.07.002 PMC5846465

[99] O. Dudchenko , S. S. Batra , A. D. Omer , S. K. Nyquist , M. Hoeger , N. C. Durand , M. S. Shamim , I. Machol , E. S. Lander , A. P. Aiden , E. L. Aiden , Science 2017, 356, 92. 28336562 10.1126/science.aal3327 PMC5635820

[100] N. C. Durand , J. T. Robinson , M. S. Shamim , I. Machol , J. P. Mesirov , E. S. Lander , E. L. Aiden , Cell Syst 2016, 3, 99. 27467250 10.1016/j.cels.2015.07.012 PMC5596920

[101] F. A. Simão , R. M. Waterhouse , P. Ioannidis , E. V. Kriventseva , E. M. Zdobnov , Bioinformatics 2015, 31, 3210. 26059717 10.1093/bioinformatics/btv351

[102] A. Shumate , S. L. Salzberg , Bioinformatics 2021, 37, 1639. 33320174 10.1093/bioinformatics/btaa1016 PMC8289374

[103] G. Pertea , M. Pertea , F1000Res 2020, 9. 10.12688/f1000research.23297.1 PMC7222033 32489650

[104] N. Patterson , A. L. Price , D. Reich , PLoS Genet. 2006, 2, 190. 10.1371/journal.pgen.0020190 PMC1713260 17194218

[105] S. Purcell , B. Neale , K. Todd‐Brown , L. Thomas , M. A. Ferreira , D. Bender , J. Maller , P. Sklar , P. I. de Bakker , M. J. Daly , P. C. Sham , Am. J. Hum. Genet. 2007, 81, 559. 17701901 10.1086/519795 PMC1950838

[106] K. Tamura , D. Peterson , N. Peterson , G. Stecher , M. Nei , S. Kumar , Mol. Biol. Evol. 2011, 28, 2731. 21546353 10.1093/molbev/msr121 PMC3203626

[107] D. H. Huson , D. Bryant , Nat. Methods 2024, 21, 1773. 39223398 10.1038/s41592-024-02406-3

[108] D. C. Jeffares , C. Jolly , M. Hoti , D. Speed , L. Shaw , C. Rallis , F. Balloux , C. Dessimoz , J. Bähler , F. J. Sedlazeck , Nat. Commun. 2017, 8, 14061. 28117401 10.1038/ncomms14061 PMC5286201

[109] P. Danecek , J. K. Bonfield , J. Liddle , J. Marshall , V. Ohan , M. O. Pollard , A. Whitwham , T. Keane , S. A. McCarthy , R. M. Davies , H. Li , Gigascience 2021, 10, giab008. 33590861 10.1093/gigascience/giab008 PMC7931819

[110] A. C. English , V. K. Menon , R. A. Gibbs , G. A. Metcalf , F. J. Sedlazeck , Genome Biol. 2022, 23, 271. 36575487 10.1186/s13059-022-02840-6 PMC9793516

[111] S. Chen , P. Krusche , E. Dolzhenko , R. M. Sherman , R. Petrovski , F. Schlesinger , M. Kirsche , D. R. Bentley , M. C. Schatz , F. J. Sedlazeck , M. A. Eberle , Genome Biol. 2019, 20, 291. 31856913 10.1186/s13059-019-1909-7 PMC6921448

[112] P. Danecek , A. Auton , G. Abecasis , C. A. Albers , E. Banks , M. A. DePristo , R. E. Handsaker , G. Lunter , G. T. Marth , S. T. Sherry , G. McVean , R. Durbin , Bioinformatics 2011, 27, 2156.21653522

[113] A. R. Quinlan , Curr Protoc Bioinformatics 2014, 47. 10.1002/0471250953.bi1112s47 PMC4213956 25199790

[114] B. Langmead , S. L. Salzberg , Nat. Methods 2012, 9, 357. 22388286 10.1038/nmeth.1923 PMC3322381

[115] H. Li , B. Handsaker , A. Wysoker , T. Fennell , J. Ruan , N. Homer , G. Marth , G. Abecasis , R. Durbin , Bioinformatics 2009, 25, 2078. 19505943 10.1093/bioinformatics/btp352 PMC2723002

[116] Y. Zhang , T. Liu , C. A. Meyer , J. Eeckhoute , D. S. Johnson , B. E. Bernstein , C. Nusbaum , R. M. Myers , M. Brown , W. Li , X. S. Liu , Genome Biol. 2008, 9, R137. 18798982 10.1186/gb-2008-9-9-r137 PMC2592715

[117] L. T. Nguyen , H. A. Schmidt , A. von Haeseler , B. Q. Minh , Mol. Biol. Evol. 2015, 32, 268. 25371430 10.1093/molbev/msu300 PMC4271533

[118] S. Kalyaanamoorthy , B. Q. Minh , T. K. F. Wong , A. von Haeseler , L. S. Jermiin , Nat. Methods 2017, 14, 587. 28481363 10.1038/nmeth.4285 PMC5453245

[119] N. Servant , N. Varoquaux , B. R. Lajoie , E. Viara , C. J. Chen , J. P. Vert , E. Heard , J. Dekker , E. Barillot , Genome Biol. 2015, 16, 259. 26619908 10.1186/s13059-015-0831-x PMC4665391

[120] O. Ursu , N. Boley , M. Taranova , Y. X. R. Wang , G. G. Yardimci , W. Stafford Noble , A. Kundaje , Bioinformatics 2018, 34, 2701. 29554289 10.1093/bioinformatics/bty164 PMC6084597

[121] M. J. Rowley , M. H. Nichols , X. Lyu , M. Ando‐Kuri , I. S. M. Rivera , K. Hermetz , P. Wang , Y. Ruan , V. G. Corces , Mol. Cell 2017, 67, 837. 28826674 10.1016/j.molcel.2017.07.022 PMC5591081

[122] E. Crane , Q. Bian , R. P. McCord , B. R. Lajoie , B. S. Wheeler , E. J. Ralston , S. Uzawa , J. Dekker , B. J. Meyer , Nature 2015, 523, 240. 26030525 10.1038/nature14450 PMC4498965

[123] A. Kaul , S. Bhattacharyya , F. Ay , Nat. Protoc. 2020, 15, 991. 31980751 10.1038/s41596-019-0273-0 PMC7451401

[124] X. Zhou , M. Stephens , Nat. Genet. 2012, 44, 821. 22706312 10.1038/ng.2310 PMC3386377

[125] H. Thorvaldsdóttir , J. T. Robinson , J. P. Mesirov , Brief Bioinform 2013, 14, 178. 22517427 10.1093/bib/bbs017 PMC3603213

[126] S. S. Dong , W. M. He , J. J. Ji , C. Zhang , Y. Guo , T. L. Yang , Brief Bioinform 2021, 22, bbaa227. 33126247 10.1093/bib/bbaa227

