# Supplementary material for: Structural Variations Associated with Adaptation and Coat Color in Qinghai‐Tibetan Plateau Cattle
Source: Adv Sci (Weinh). 2025 Jun 5;12(31):e03258. doi: 10.1002/advs.202503258 (PMC12376619; doi:10.1002/advs.202503258)
Supplement: Supplementary file 1 — Supporting Information [file ADVS-12-e03258-s002.docx]

**Structural Variations Associated with Adaptation and Coat Color in Qinghai-Tibetan Plateau Cattle**

*Xiaoting Xia, Fuwen Wang, Xiaoyu Luo, Shuang Li, Yang Lyu, Yining Zheng, Zhijie Ma, Kaixing Qu, Rende Song, Jianyong Liu, Jicai Zhang, Basang Wangdui, Basang Zhuzha, Suolang Quji, Li Zhao, Silang Wangmu, Ciren Luobu, Nima Cangjue, Danzeng Luosang, Suolang Sizhu, Haijian Cheng, Ruizhe Li, Zhipeng Wu, Ruihua Dang, Yongzhen Huang, Xianyong Lan, Luohao Xu, Haifei Hu, WaiYee Low, Zhuqing Zheng, Yu Wang, Yuanpeng Gao, Lu Deng, Johannes A. Lenstra, Jianlin Han, Xueyi Yang, Wenfa Lyu*, Bizhi Huang*, Chuzhao Lei*, Ningbo Chen**


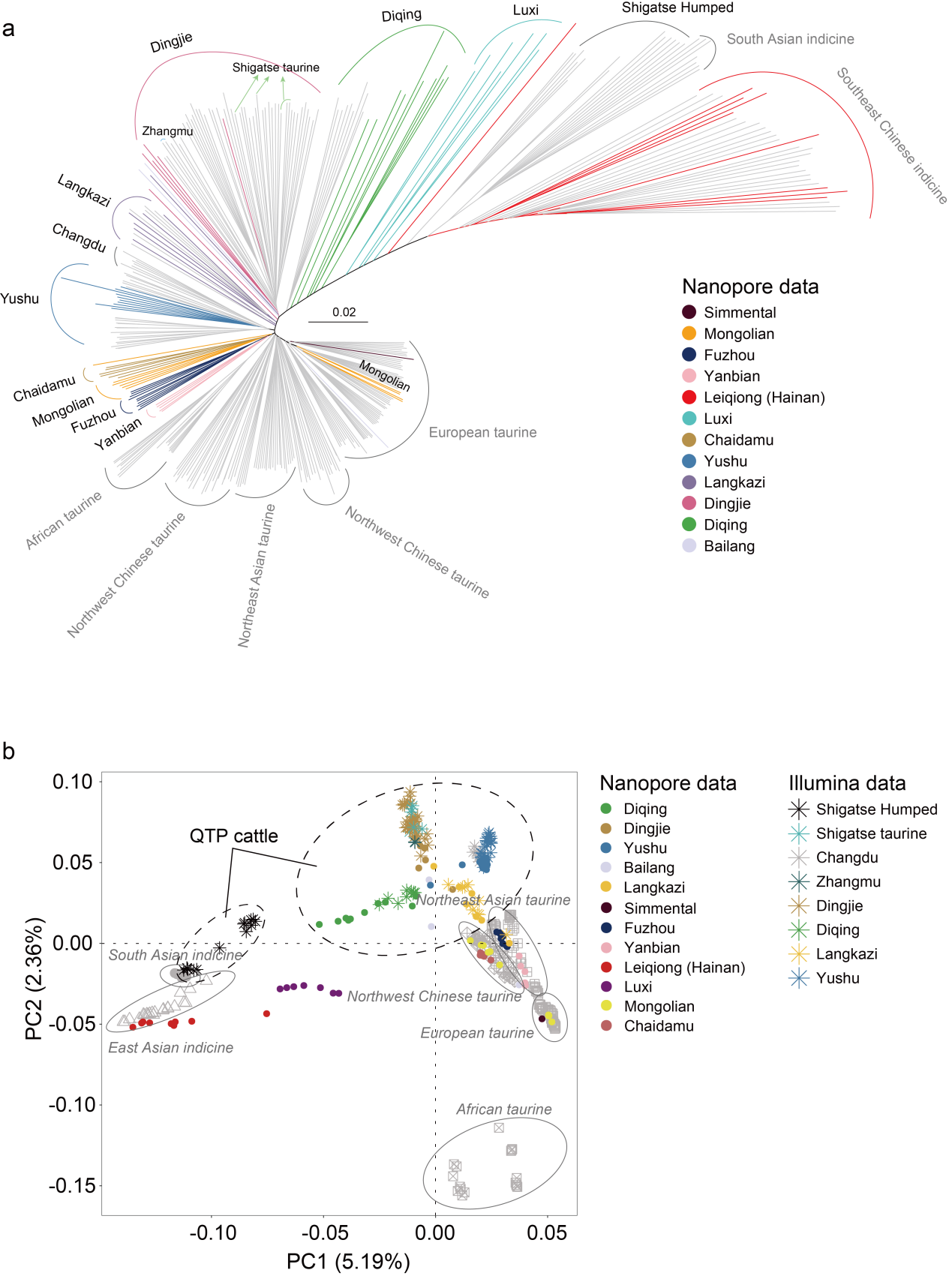


**Figure S1**. Phylogenetic tree and principal component constructed via whole-genome autosomal SNP data from 365 cattle. a) Neighbor-joining tree of the genome sequences used in this study. The colored branches represent 84 LRS data points, and the gray branches represent 281 SRS data points. b) Principal component (PC) analysis of 365 individuals. The colored dots represent the data from 84 LRS data, the colored snowflakes represent the data from 112 SRS data from QTP cattle, and the gray shapes represent the SRS data from cattle in other regions.


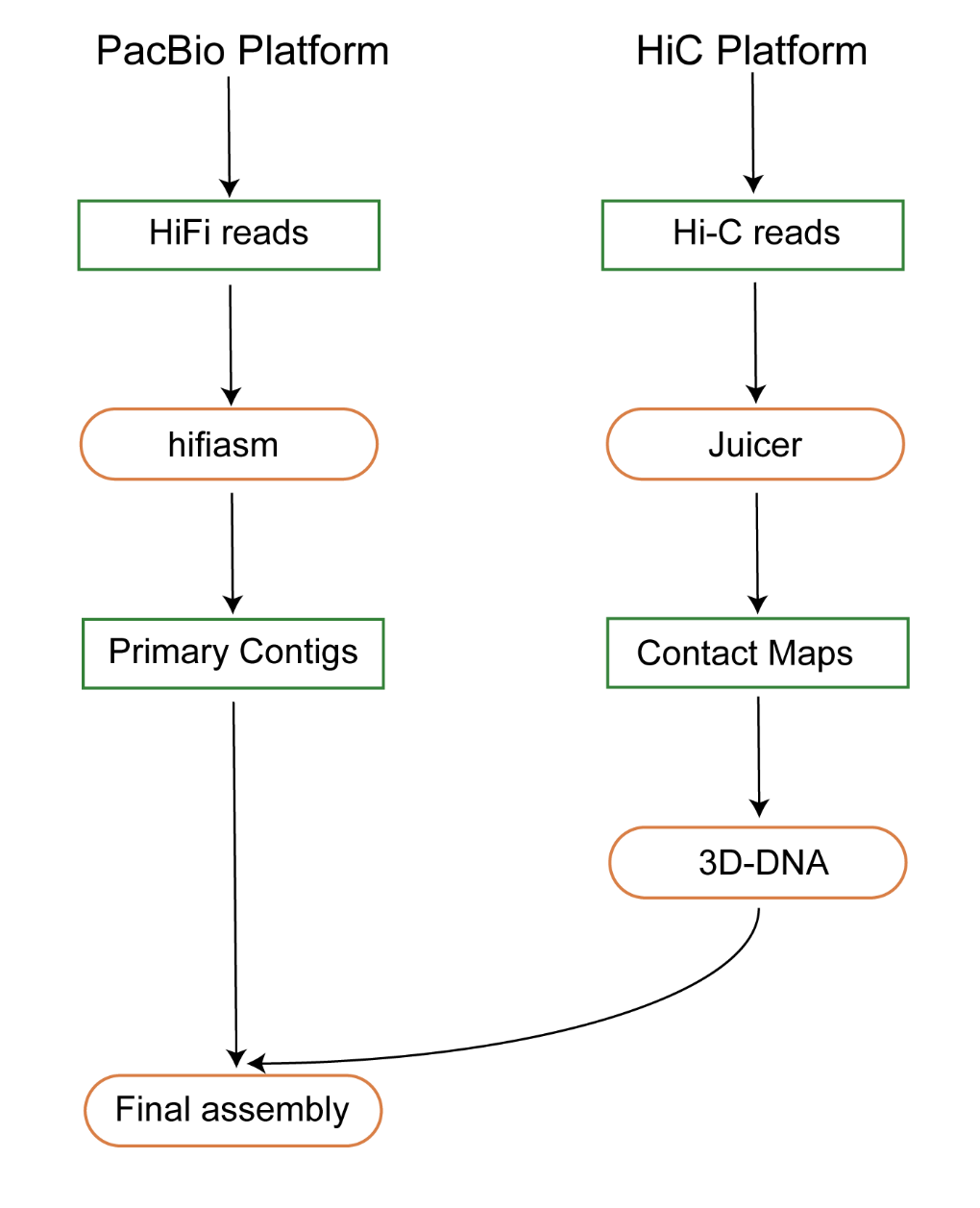


**Figure S2.** Flowchart for assembly of the Tibetan_v1 genome.


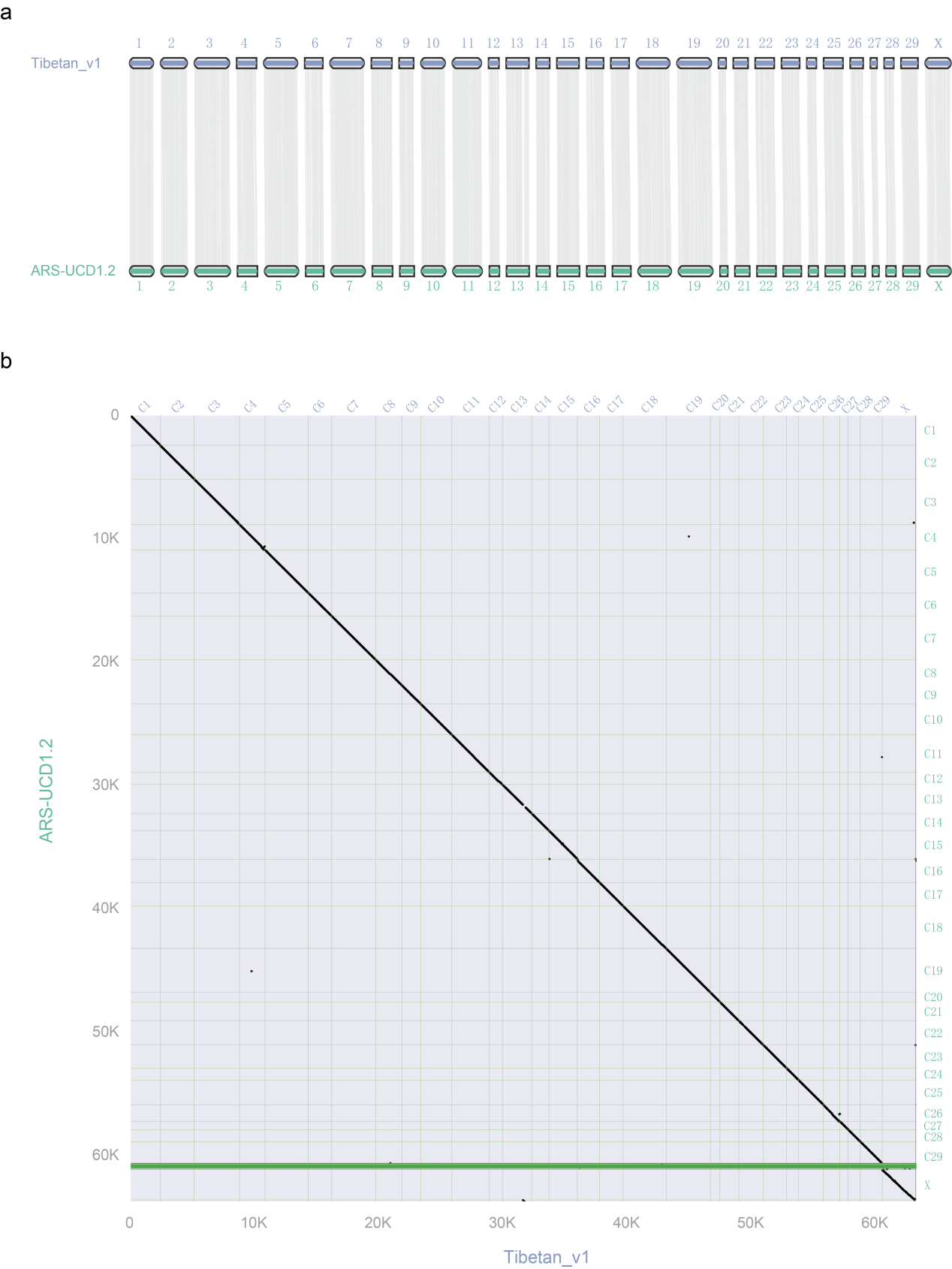


**Figure S3.** Collinear comparison of the Tibetan cattle genome and a reference cattle genome (ARS-UCD1.2). a) Genome synteny analysis between Tibetan_v1 and ARS-UCD1.2. b) Dot plots illustrating collinear blocks (continuous points) of the ARS-UCD1.2 (left) and Tibetan_v1 (right) genomes ordered by chromosome.


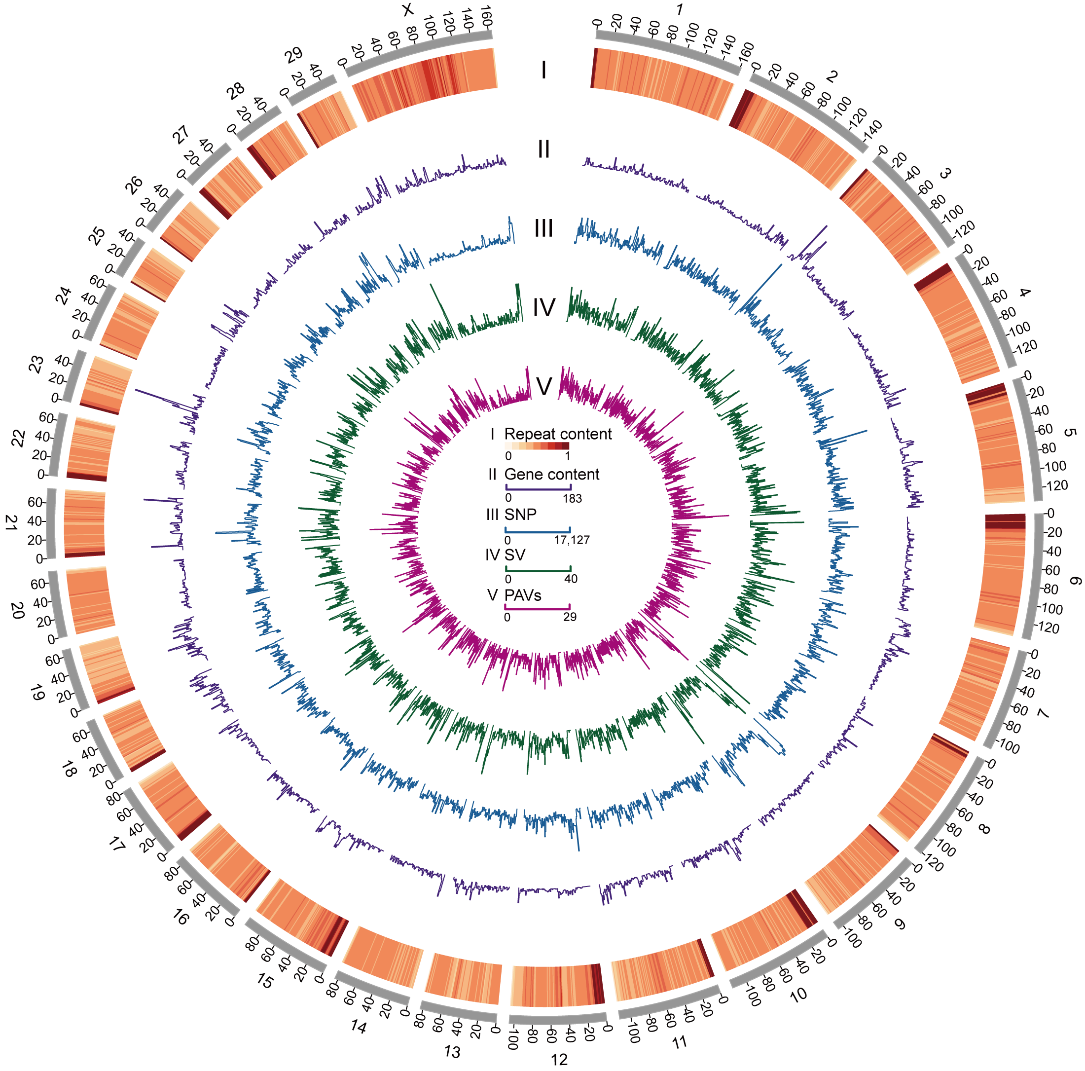


**Figure S4.** Circos view of the genome assembly of the Tibetan_v1 genome. Track I represents the repeat content, track II represents the gene density, track III represents the SNP density, track IV represents the SV density, and track V represents the PAV density.


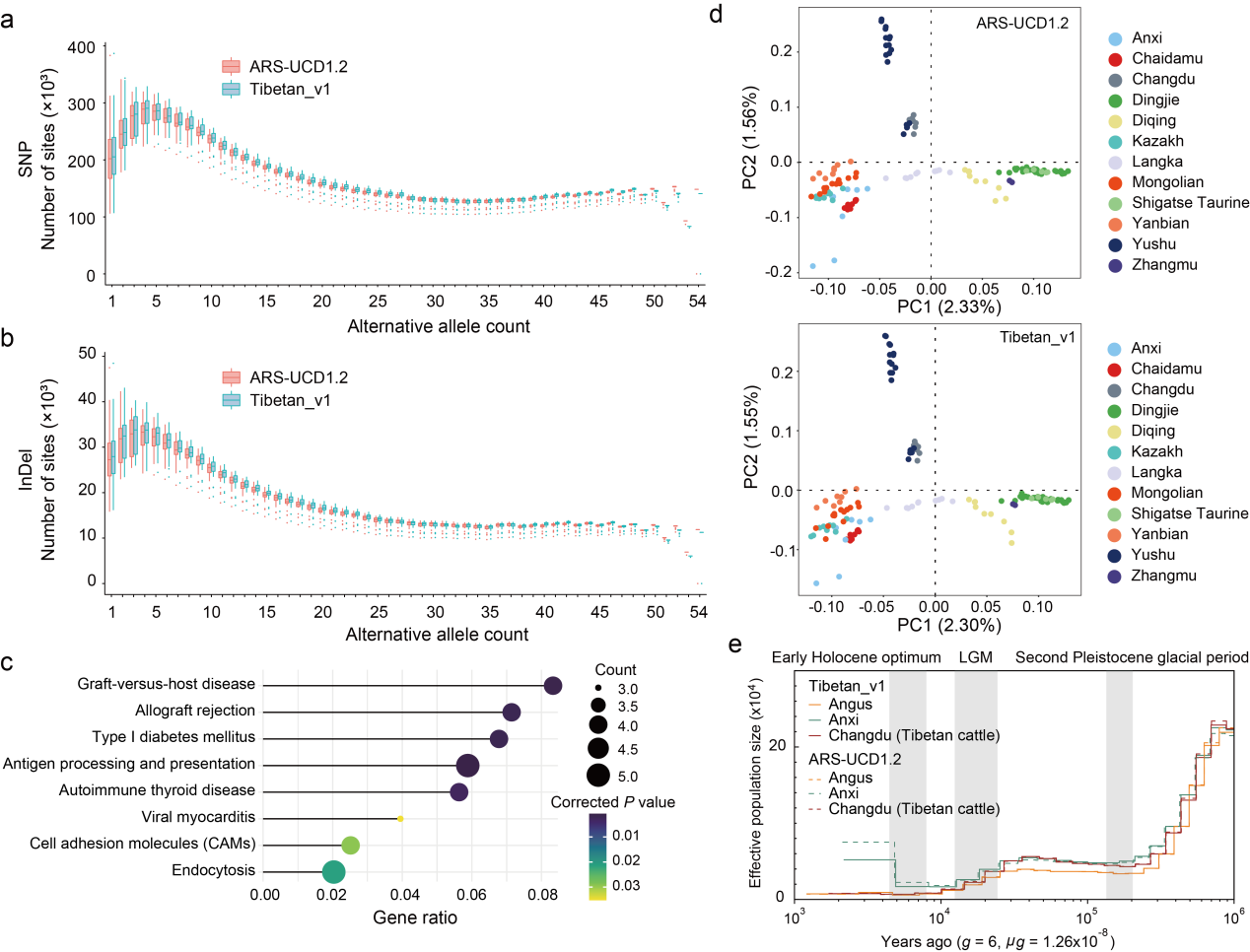


**Figure S5.** Lineage-specific assemblies reveal fine-scale population genetic properties. Alternative allele counts of SNPs (a) and InDels (b) for 27 Dingjie cattle (54 haplotypes) with short-read sequencing based on different reference assemblies (ARS-UCD1.2 and Tibetan_v1). Each dot represents an individual genome. **c**) Enrichment pathway analysis for the “converted” multiallelic variants. **d**) PCA plots of 11 cattle populations in northern China based on Tibetan_v1 and ARS-UCD1.2. **e**) Changes in the effective population size over time based on the SNPs from different assemblies. LGM, Last Glacial Maximum.


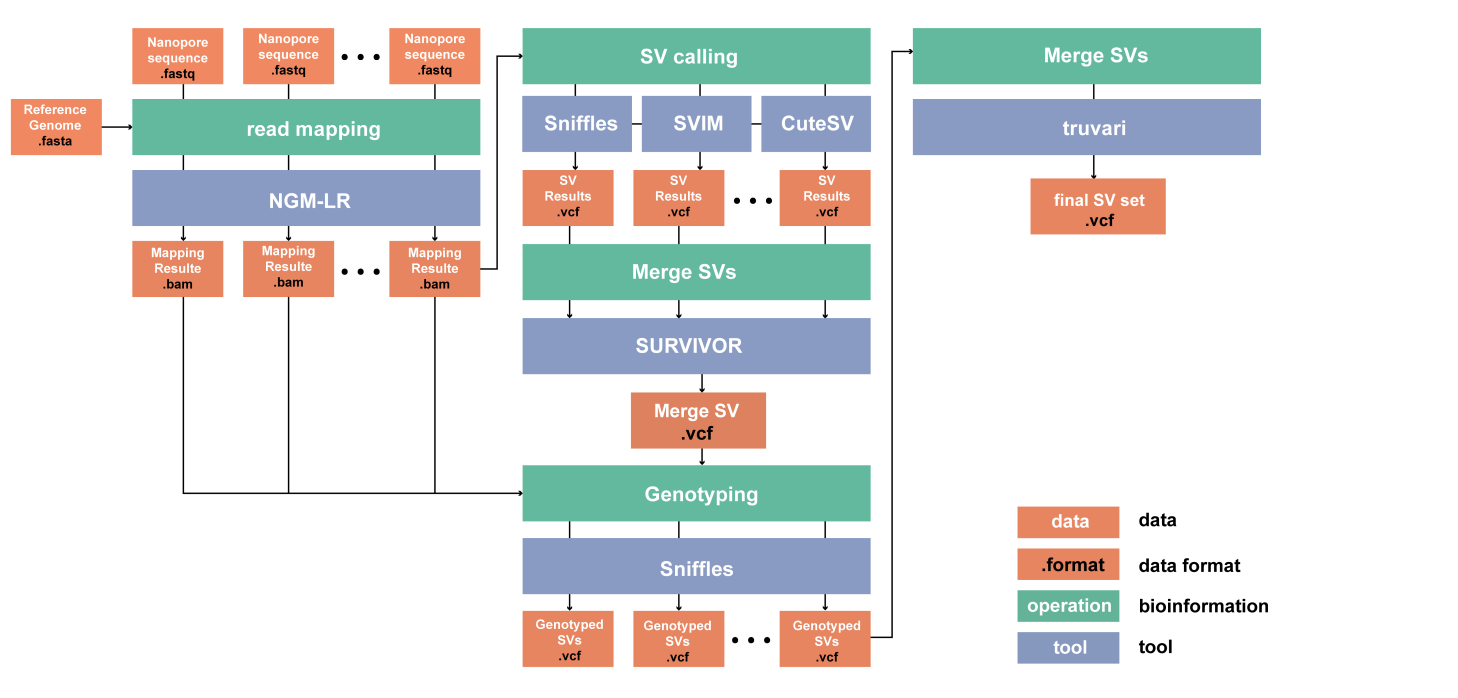


**Figure S6.** Flowchart for calling SVs.


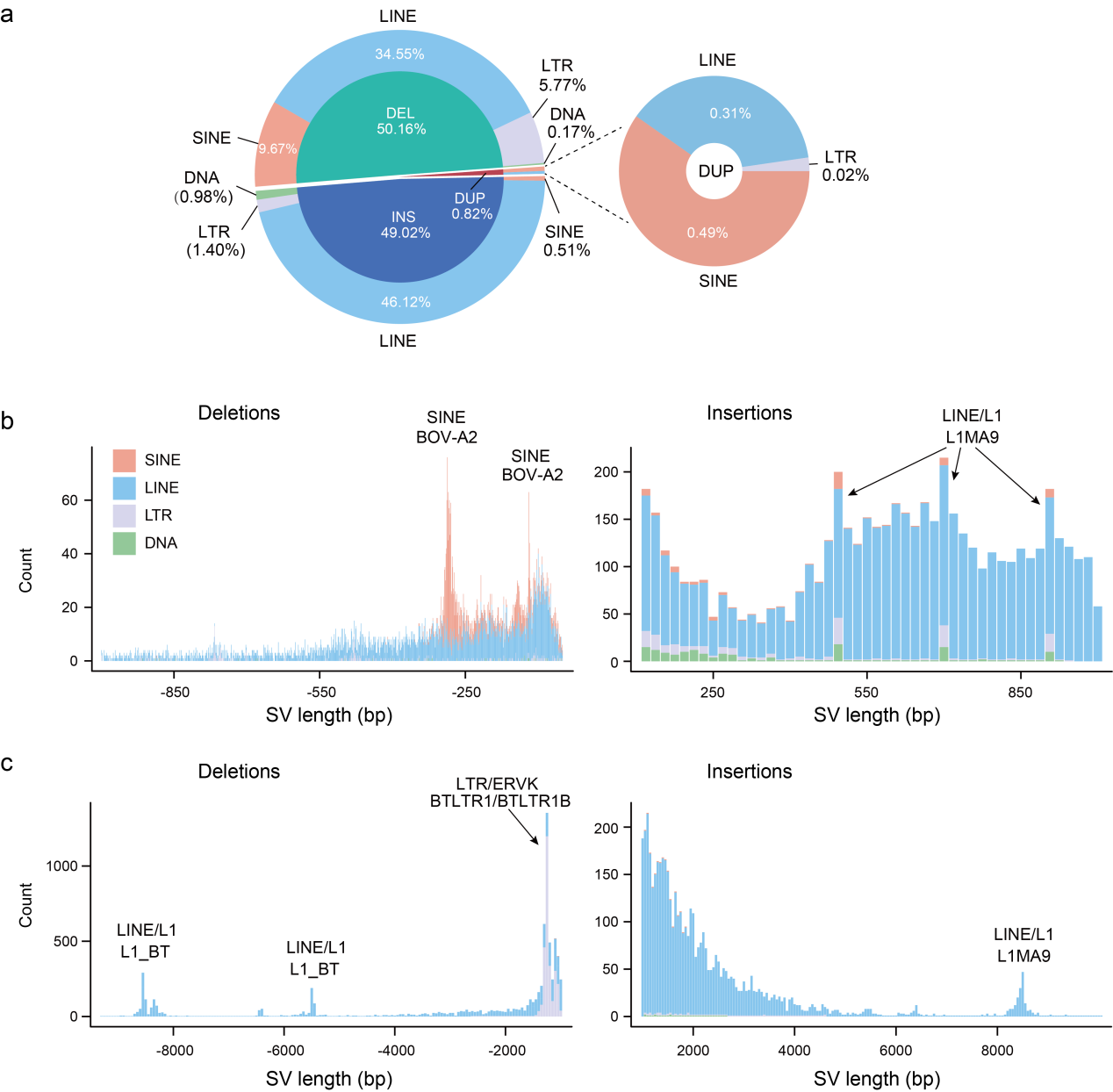


**Figure S7.** The composition of TE-derived SVs identified in 84 cattle. a) Percentages of four major TE types (DNA transposons, SINEs, LINEs, and LTRs) in insertions (INSs), deletions (DELs) and duplications (DUPs). (b-c), Length distributions of different types of SVs. Size distributions of INSs (positive values) and DELs (negative values), parsed by type (color scheme as in b).


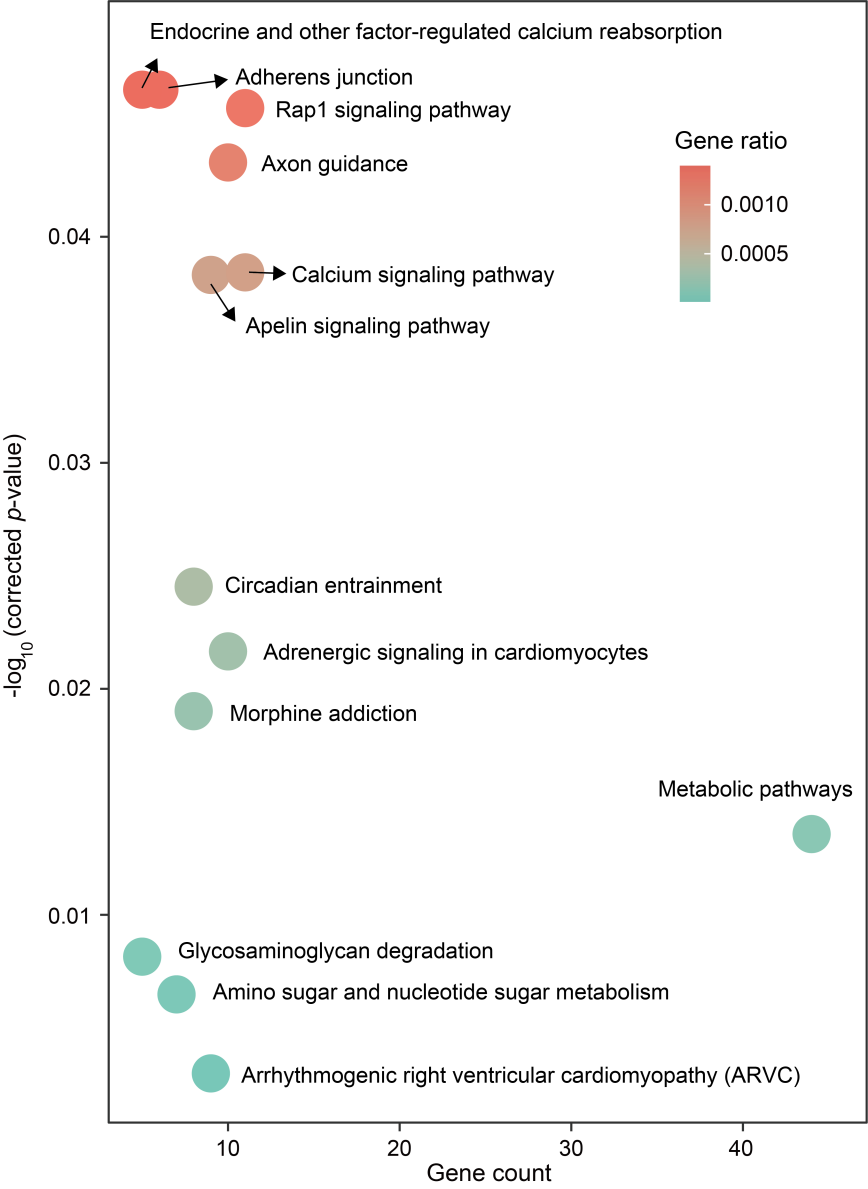


**Figure S8.** KEGG enrichment analysis of the candidate genes of the identified *d_i_*-SVs.


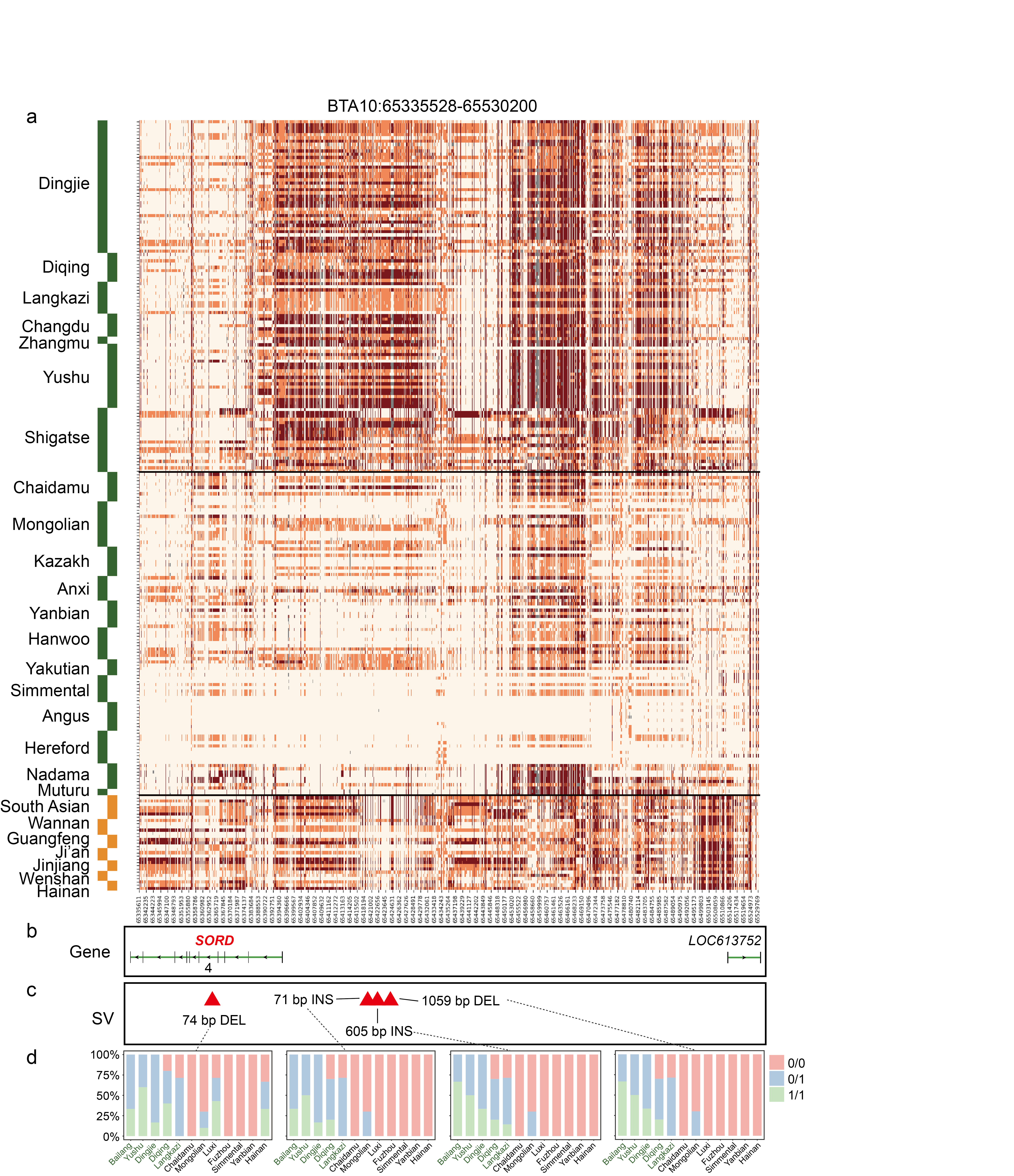


**Figure S9.** The pattern of SNP genotypes in the *SORD* and *LOC613752* regions. a) Heatmap showing the haplotype structure of *SORD*. The rows represent individuals, and the columns represent the positions of SNPs in the genomes. b) Gene distribution. c) SV distribution. d) Genotype frequency distributions of the four SVs in 84 LRS genomes.


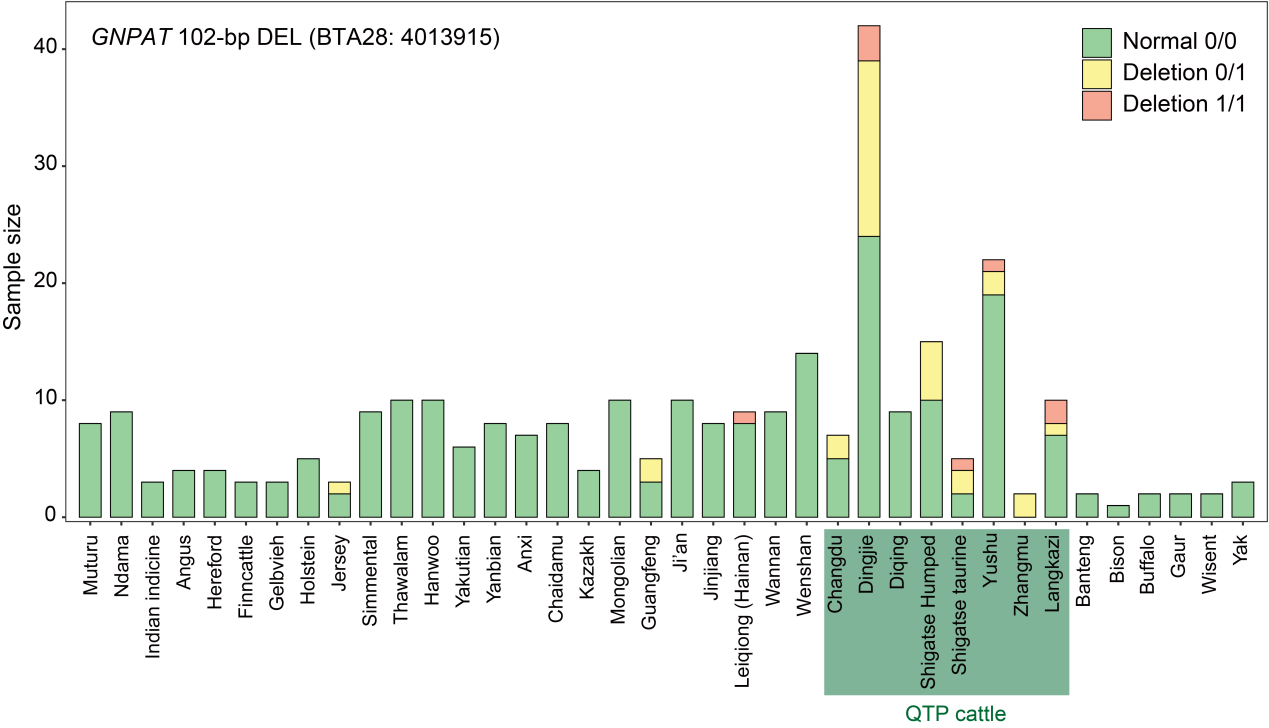


**Figure S10.** The allele frequency of a 102-bp DEL in the *GNPAT* gene detected in 293 SRS cattle samples, comprising 281 cattle and 12 wild bovine species (banteng, bison, buffalo, gaur, wisent, and yak).


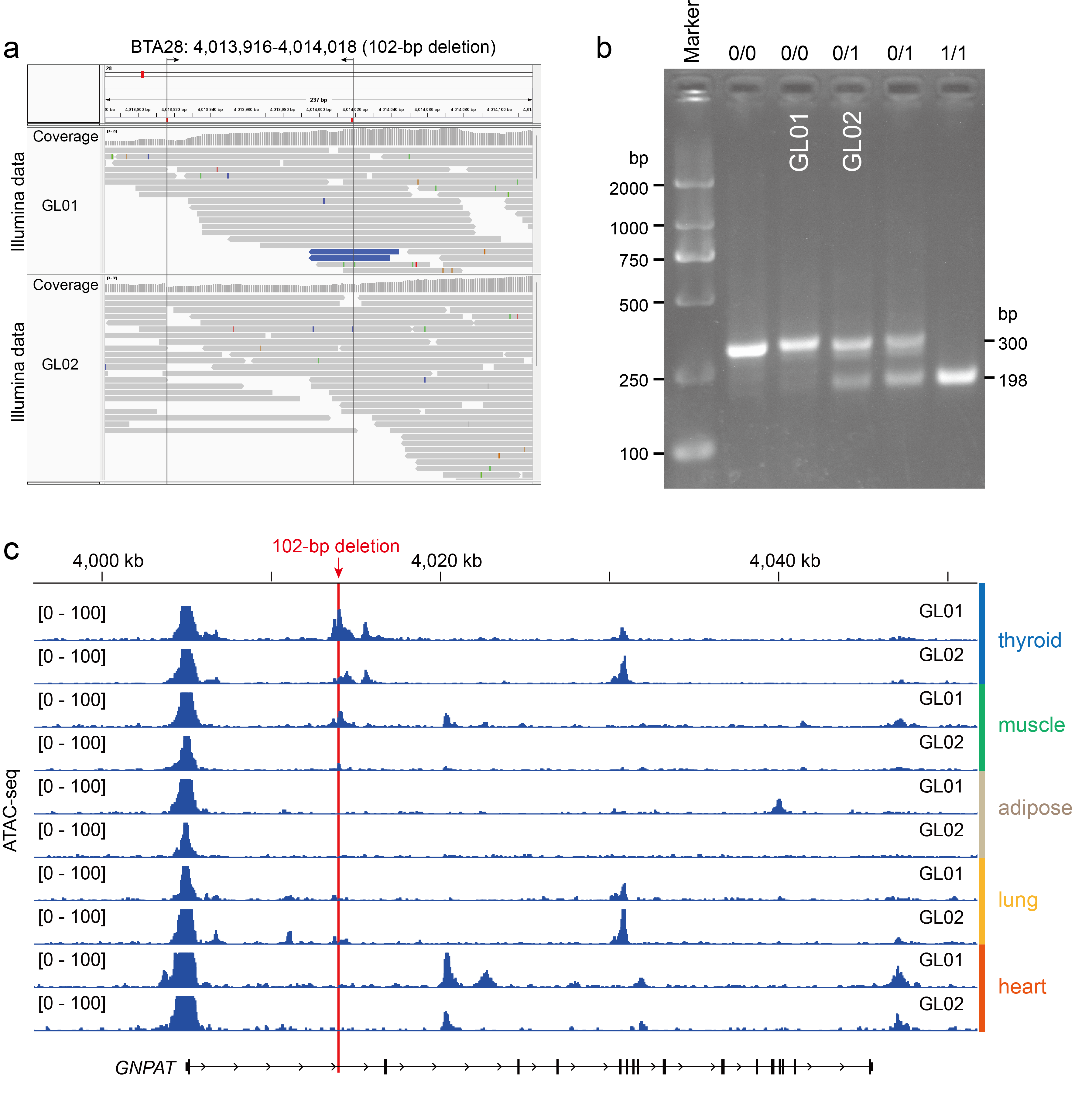


**Figure S11**. Genetic and epigenetic characterization of the 102-bp deletion in Tibetan cattle. a) IGV screenshot of the 102-bp deletion (BTA28: 4,013,916-4,014,018) in two Tibetan cattle (GL01 and GL02). b) PCR validation of the 102-bp deletion in GL01 and GL02. c) The blue tracks represent the ATAC-seq data from five tissues (thyroid, muscle, adipose, lung, and heart) in Tibetan cattle samples GL01 and GL02. The red line shows the 102-bp DEL (BTA28:4013916-4014018) in GNPAT, located within chromatin accessibility signals.


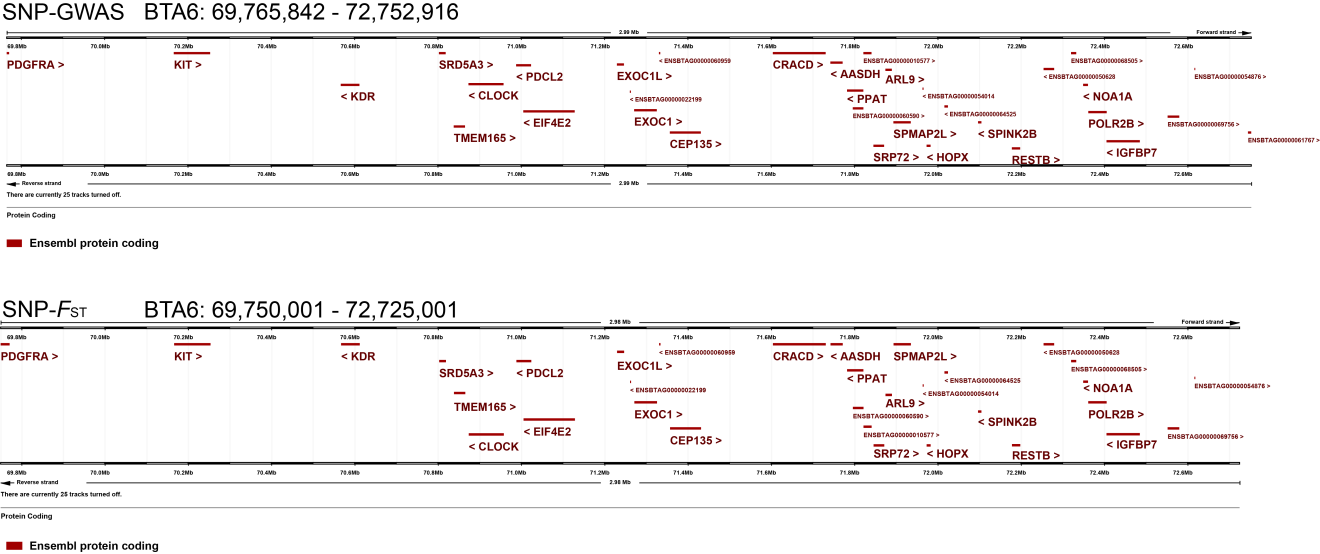


**Figure S12.** Gene distribution diagram of the SNP-GWAS (top) and the SNP-*F*_ST_ signaling region (bottom).


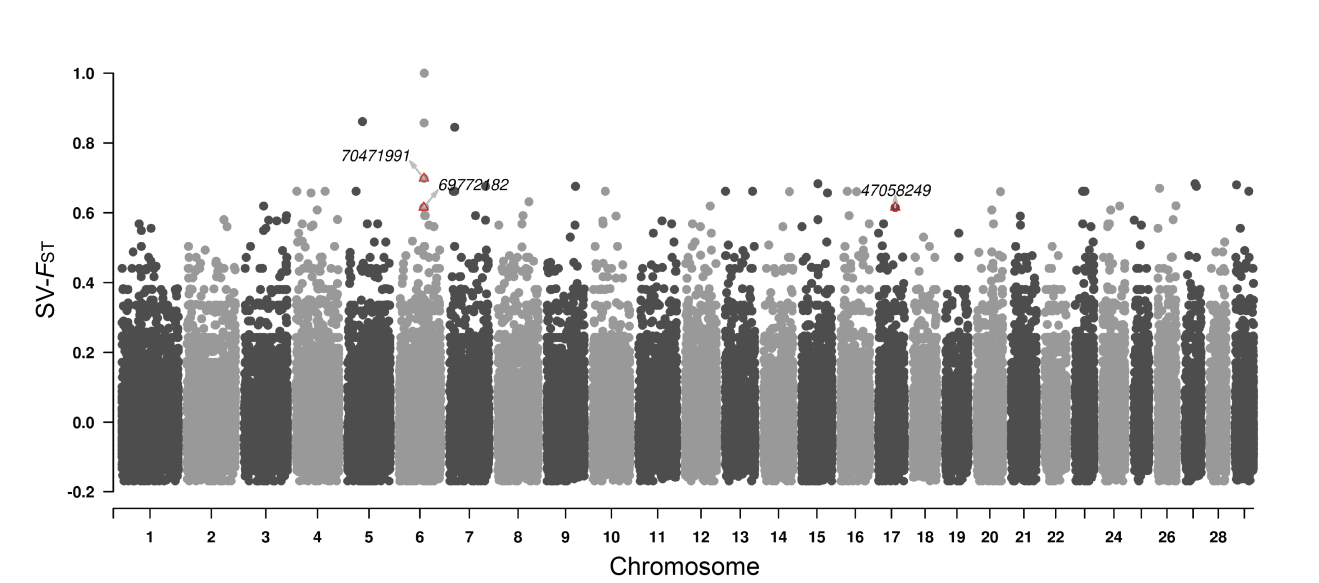


**Figure S13.** Manhattan plot of SV-*F*_ST_ values calculated between five gray and 11 non-gray QTP cattle. The red triangle indicates an inversion (BTA6:69,772,182-71,772,197) and a translocation (BTA6:70,471,991-17:46,961,621) on BTA6, as well as a translocation (BTA17:47,058,249-BTA6:69,764,591) on BTA17.


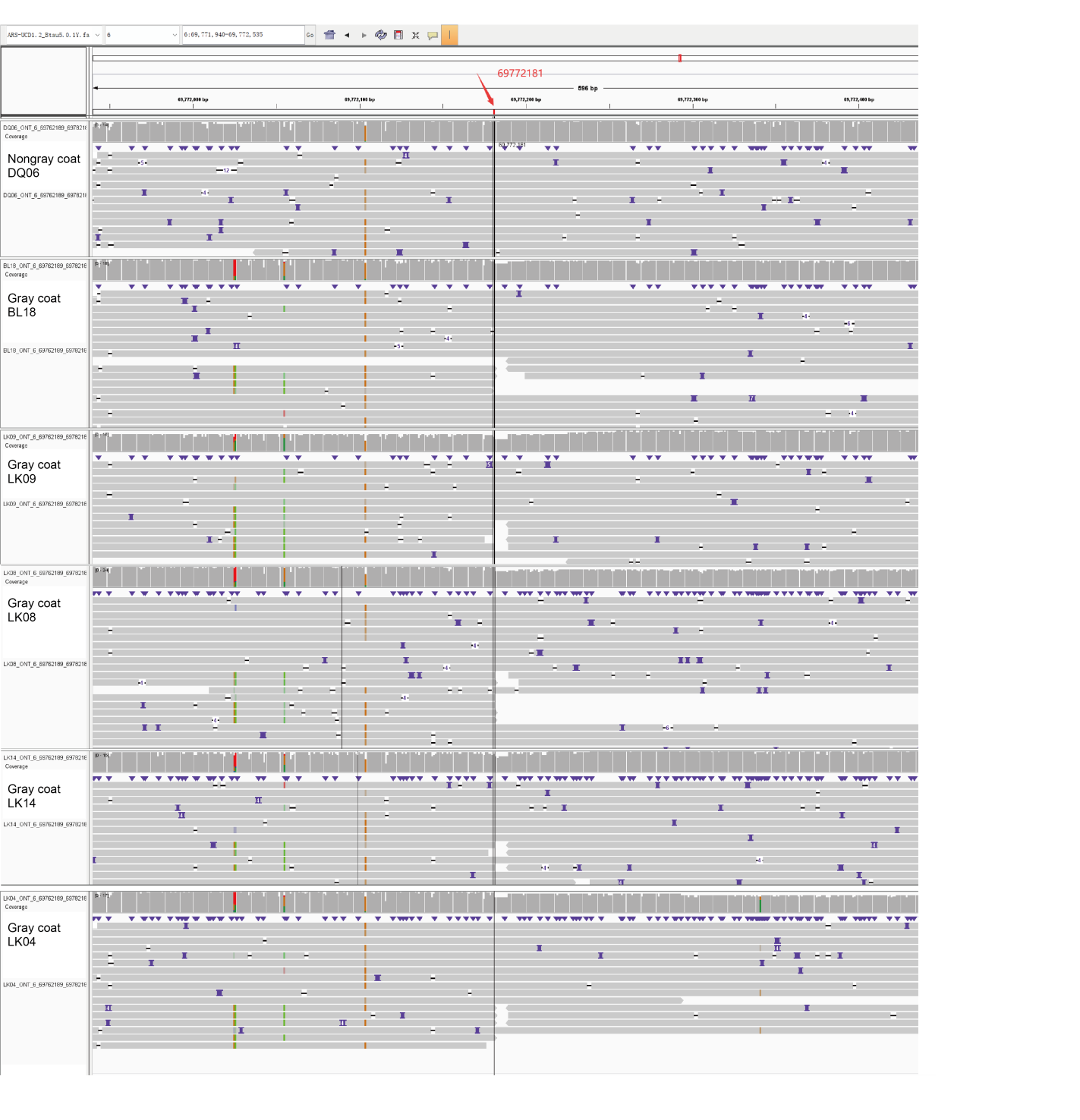


**Figure S14.** IGV screenshot of the 2-Mb heterozygous INV in five gray cattle (BL18, LK04, LK08, LK09 and LK14) and one non-gray cattle (DQ06).
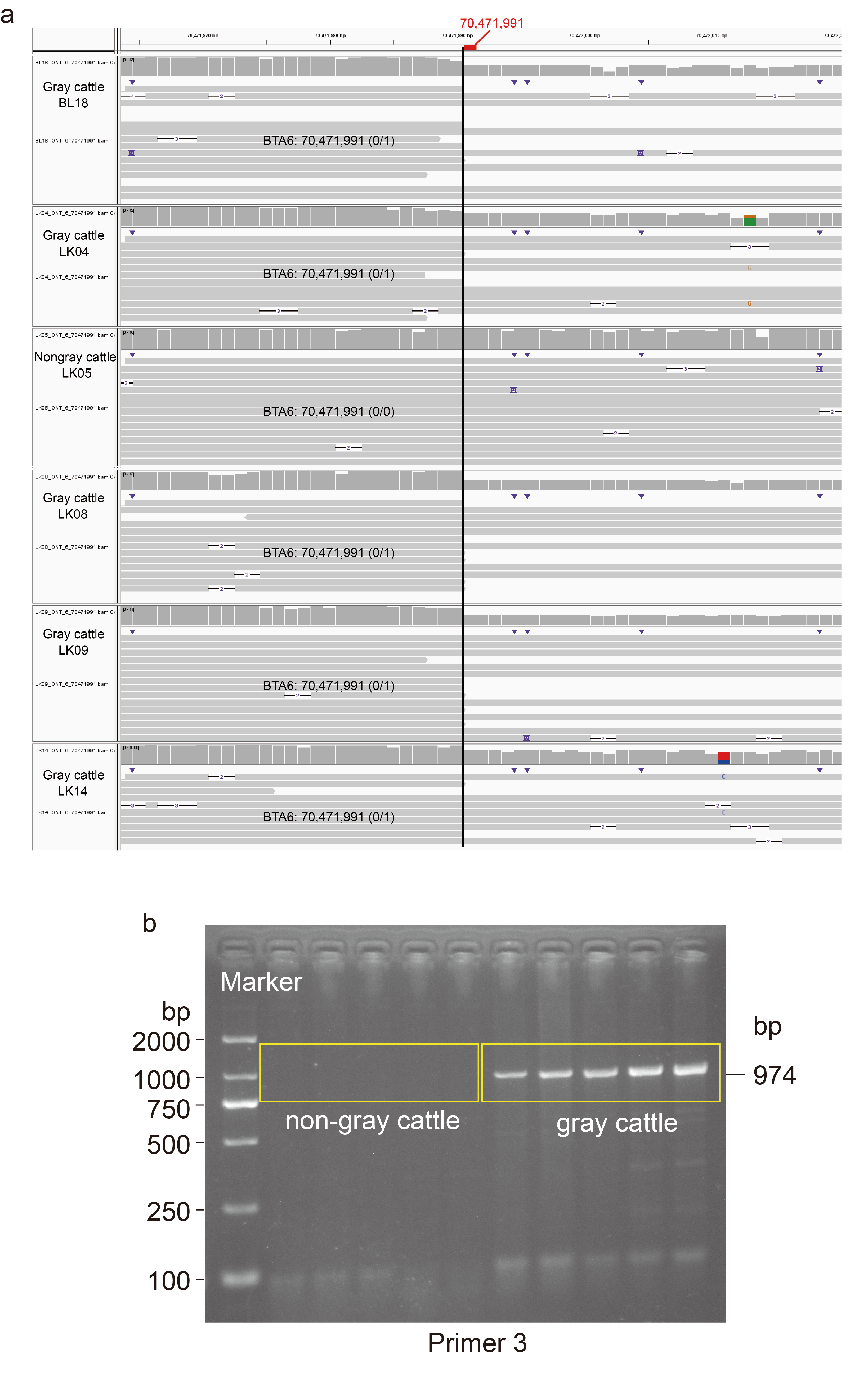


**Figure S15**. Molecular validation of a translocation (BTA6:70,471,991) in gray cattle. a) IGV screenshot of the BTA6 TRA (BTA6:70,471,991) in five gray cattle (BL18, LK04, LK08, LK09 and LK14) and one non-gray cattle (LK05). b) PCR validation of the TRA (BTA6:70,471,991). We designed PCR primer at the breakpoint of the translocation (Primer3, BTA6: 70,471,991) and validated them in gray and non-gray cattle.


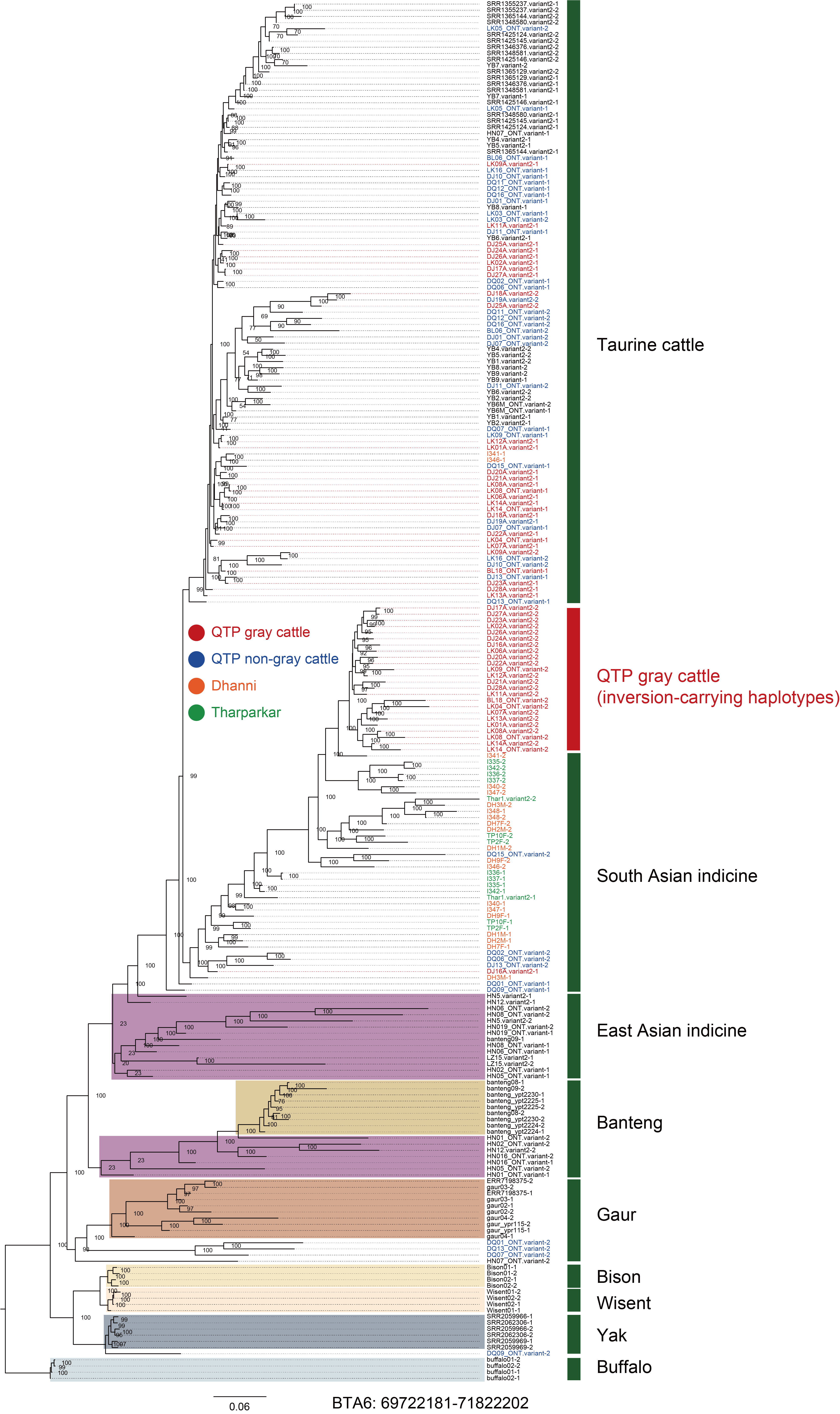


**Figure S16**. Construction of the neighbor-joining (NJ) phylogenetic tree based on 2-Mb inversion haplotype sequences. Among the 113 individuals included in the tree construction, the sample set comprised 28 gray cattle, 19 QTP non-gray cattle, along with control groups including European taurine cattle, East Asian taurine cattle, East Asian indicine cattle, South Asian indicine cattle, banteng, gaur, bison, wisent, yak, and buffalo.


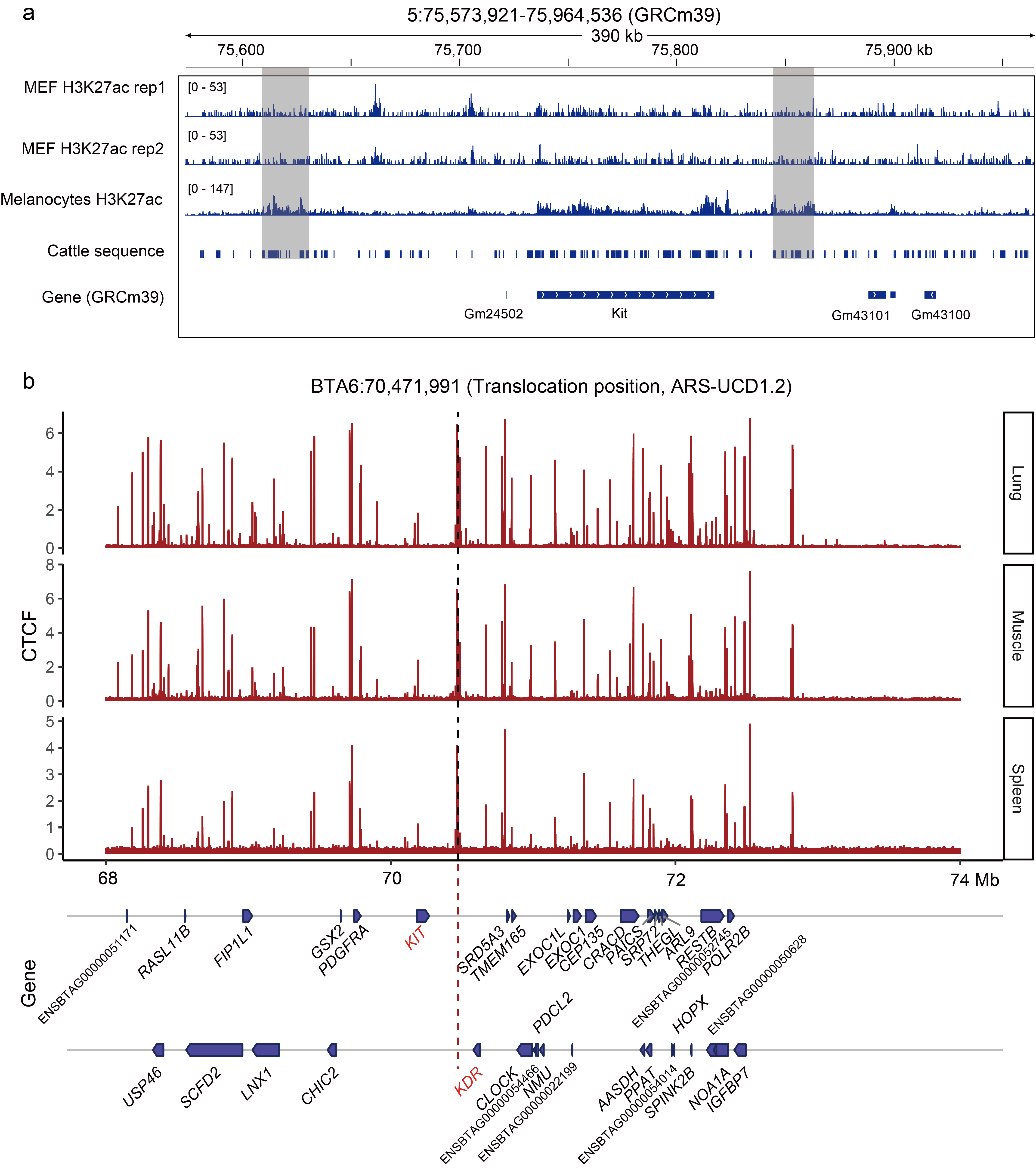


**Figure S17**. **Epigenetic conservation and chromatin architecture of flanking regions of the *KIT* gene in cattle and mice.** a) A consistency comparison was conducted between the sequences surrounding the *KIT* gene in cattle and mice. The cattle genomic region spanning BTA6:69,000,000-79,000,000 was mapped to the mouse genome (GRCm39) with an 80% match using LiftOver software. H3K27ac data from wild-type mice embryonic fibroblasts (MEFs) and melanocytes were obtained from a previous study ^1^. The shaded regions indicate enhancers flanking the *KIT* gene in mice melanocytes, as reported in the same study ^1^. b) The CTCF track from three cattle tissue (lung, muscle and spleen), and the dotted line is the position of the translocation (BTA6:70,471,991). The bottom panel shows an annotation map of genes located on BTA6 between positions 68–74 Mb in the ARS-UCD1.2 cattle genome from the Ensembl database. The source of CTCF data is provided in Supplementary table S14.


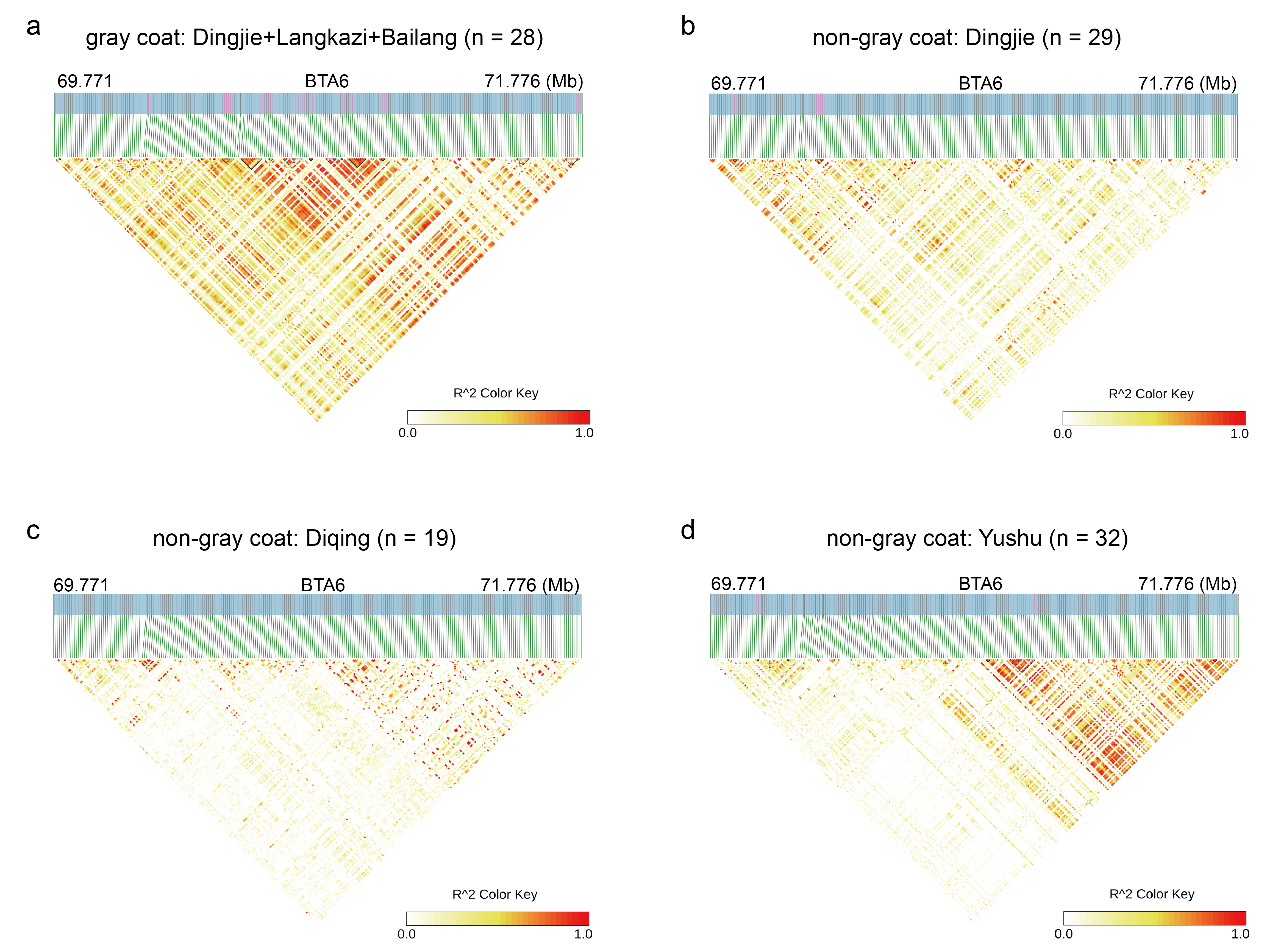


**Figure S18**. Linkage disequilibrium based on SNPs between gray and non-gray cattle in the inversion region (BTA6:69.77-71.78 Mb). Disequilibrium is measured as *r*^2^. a) Gray cattle group: comprising 28 samples from Dingjie (n = 13), Langkazi (n = 14), and Bailang cattle (n = 1) in the Qinghai-Tibetan Plateau. b-d) Non-gray cattle group: consisting of Dingjie (b, n = 29), Diqing (c, n = 19), and Yushu cattle (d, n = 32) from the Qinghai-Tibetan Plateau.


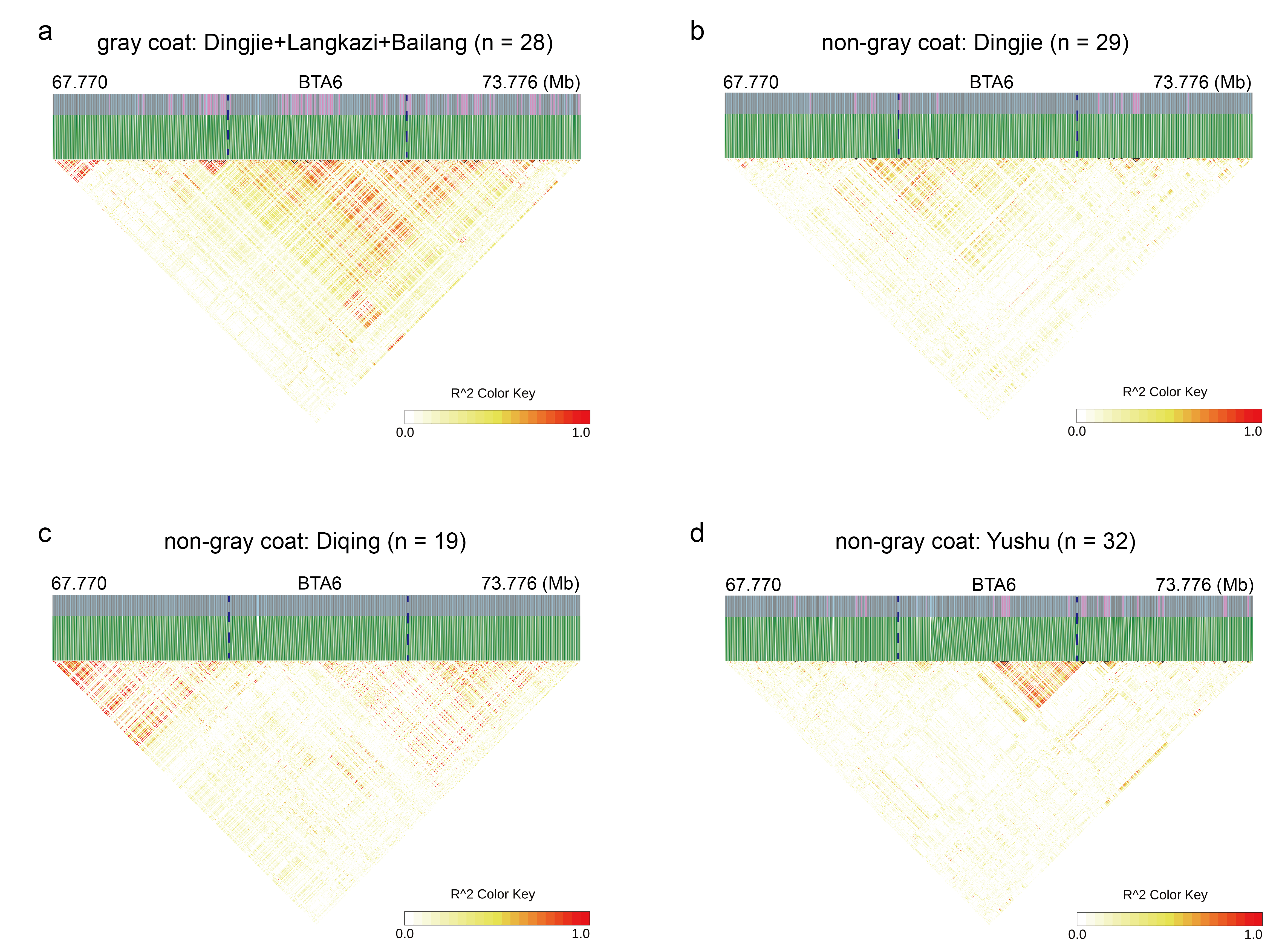


**Figure S19**. Linkage disequilibrium based on SNPs between gray and non-gray cattle in the extend inversion region (BTA6:67.77-73.78 Mb). Inversion breakpoints are depicted as vertical dashed blue lines. Disequilibrium is measured as *r*^2^. a) Gray cattle group: comprising 28 samples from Dingjie (n = 13), Langkazi (n = 14), and Bailang cattle (n = 1) in the Qinghai-Tibetan Plateau. b-d) Non-gray cattle group: consisting of Dingjie (b, n = 29), Diqing (c, n = 19), and Yushu cattle (d, n = 32) from the Qinghai-Tibetan Plateau.


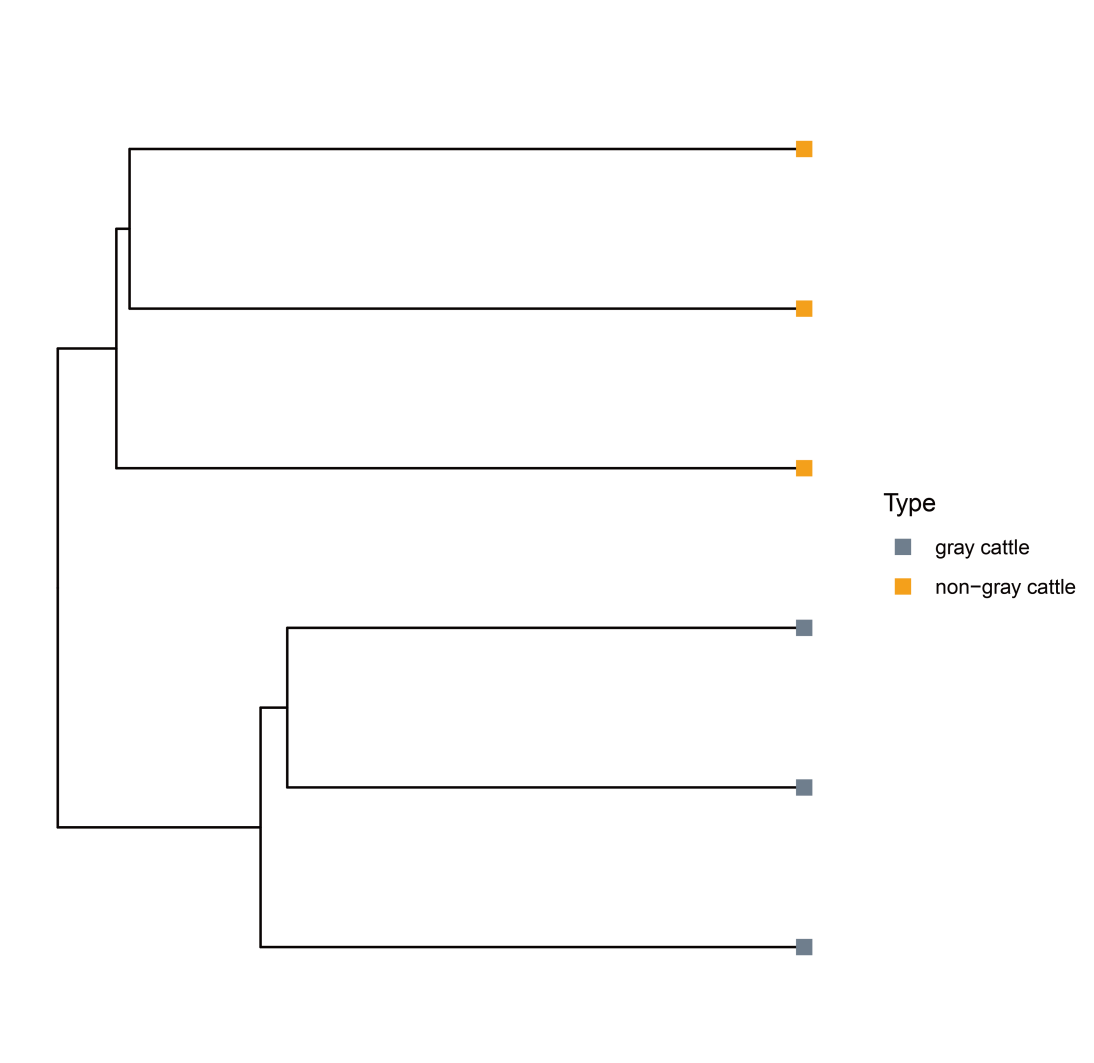


**Figure S20.** Hierarchical clustering of correlation coefficients between Hi−C library samples.

**
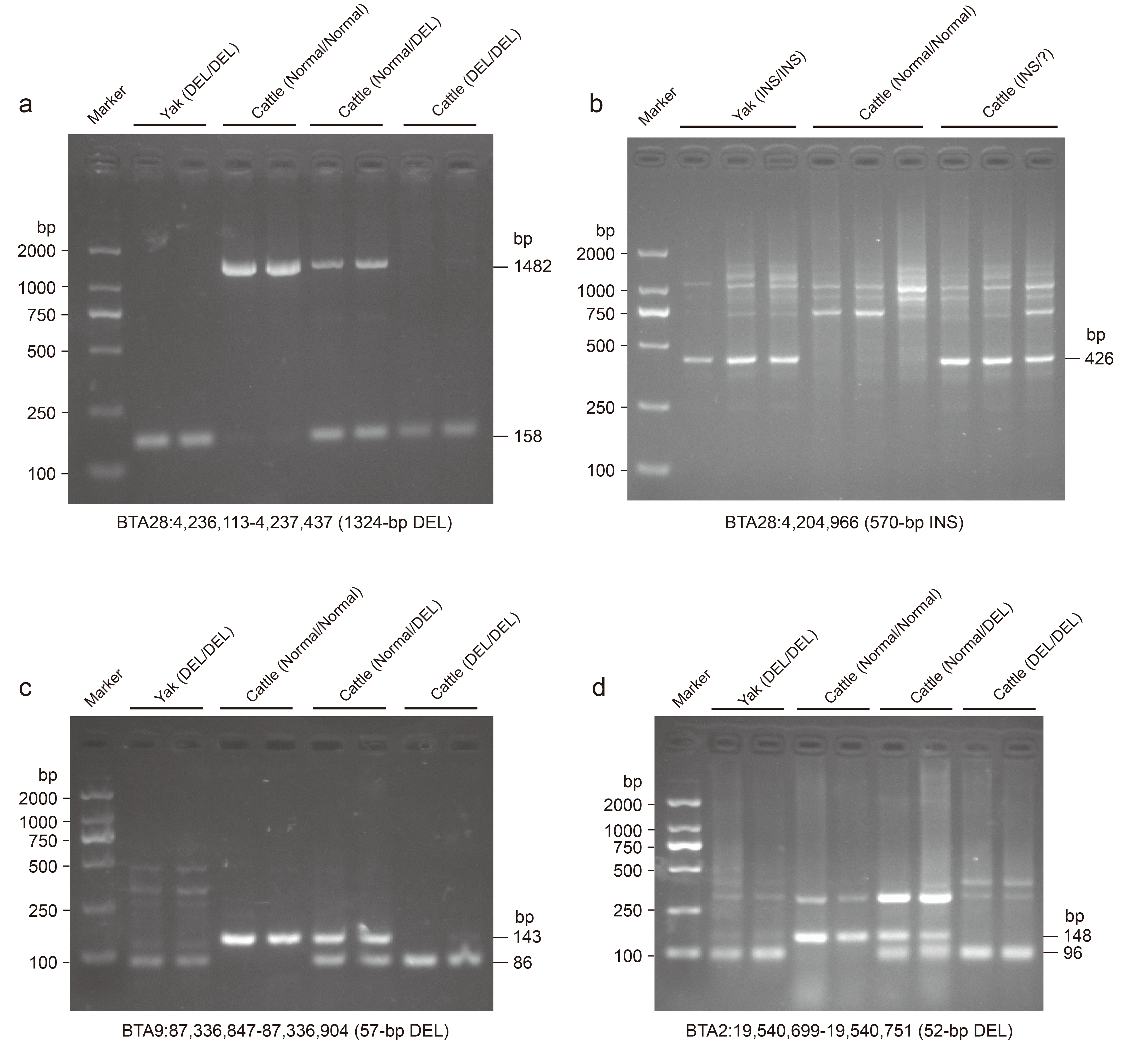
**

**Figure S21**. Confirmation of four SVs introgressed from yak into QTP cattle through PCR amplification and agarose gel electrophoresis.

Reference

1. Kabirova, E. *et al.* TAD border deletion at the Kit locus causes tissue-specific ectopic activation of a neighboring gene. *Nat Commun* **15**, 4521 (2024).
